# Clarifying the Relationship between the Lithium Deposition Coverage and Microstructure in Lithium Metal Batteries

**DOI:** 10.1021/jacs.2c08849

**Published:** 2022-11-23

**Authors:** Qidi Wang, Chenglong Zhao, Shuwei Wang, Jianlin Wang, Ming Liu, Swapna Ganapathy, Xuedong Bai, Baohua Li, Marnix Wagemaker

**Affiliations:** † Department of Radiation Science and Technology, Delft University of Technology, Mekelweg 15, Delft2629JB, The Netherlands; ‡ Shenzhen Key Laboratory on Power Battery Safety and Shenzhen Geim Graphene Center, School of Shenzhen International Graduate, Tsinghua University, Guangdong518055, China; § State Key Laboratory for Surface Physics, Institute of Physics, Chinese Academy of Sciences, Beijing100190, China

## Abstract

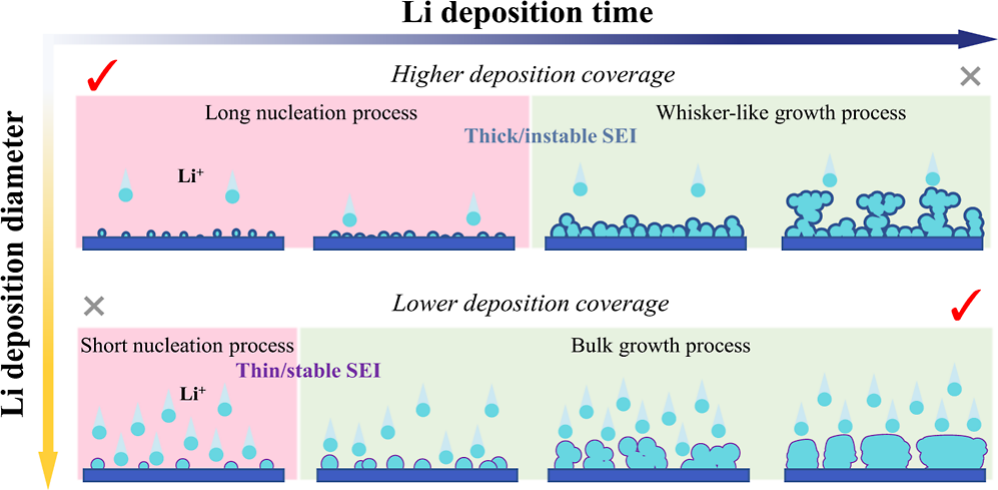

Improving the reversibility of lithium metal batteries is one of the challenges in current battery research. This requires better fundamental understanding of the evolution of the lithium deposition morphology, which is very complex due to the various parameters involved in different systems. Here, we clarify the fundamental origins of lithium deposition coverage in achieving highly reversible and compact lithium deposits, providing a comprehensive picture in the relationship between the lithium microstructure and solid electrolyte interphase (SEI) for lithium metal batteries. Systematic variation of the salt concentration offers a framework that brings forward the different aspects that play a role in cycling reversibility. Higher nucleation densities are formed in lower concentration electrolytes, which have the advantage of higher lithium deposition coverage; however, it goes along with the formation of an organic-rich instable SEI which is unfavorable for the reversibility during (dis)charging. On the other hand, the growth of large deposits benefiting from the formation of an inorganic-rich stable SEI is observed in higher concentration electrolytes, but the initial small nucleation density prevents full coverage of the current collector, thus compromising the plated lithium metal density. Taking advantages of the paradox, a nanostructured substrate is rationally applied, which increases the nucleation density realizing a higher deposition coverage and thus more compact plating at intermediate concentration (∼1.0 M) electrolytes, leading to extended reversible cycling of batteries.

## Introduction

Since the appearance of first commercial lithium (Li)-ion batteries (LIBs) in the early 1990s, they have been widely used to power mobile electronic devices.^1,2^ The increase in energy density and the reduction in the price of LIBs have enabled the introduction of electrical vehicles; however, to push this further, higher energy densities are required to increase the driving range. In this context, Li metal is interesting, having the highest theoretical specific capacity (3860 mA h g^–1^) and lowest potential (−3.04 V vs the standard hydrogen electrode). It is therefore intensively studied to break the specific energy bottleneck of current LIBs.^3,4^ However, even after decades of intensive research, its poor electrochemical reversibility and consequentially short cycle life remain the challenges that prevent commercialization.^5−7^

It is widely accepted that the electrochemical reversibility is correlated with the evolution of Li metal morphology and solid electrolyte interphase (SEI) on the Li metal surface. Heterogeneous Li metal plating results in high-surface-area “mossy” or “whisker-like” morphologies, where the high Fermi energy level of Li metal causes irreversible reactions with the electrolyte that generate the SEI, which leads to the loss of active Li, both as SEI species and as “dead” Li, the latter referring to the formation of electronically disconnected Li metal particles.^8−10^ Ideally, Li metal is electrochemically plated as a compact layer, having a small interface area with the electrolyte, where a flexible and stable SEI prevents further electrolyte decomposition. To achieve this, many strategies have been reported, which include external strategies such as applying the pressure^11−14^ and increasing the temperature,^15−18^ aiming at physically generating a more compact deposition morphology. On the other hand, internal strategies are being investigated, in which formulating electrolyte compositions aims at tuning both Li metal morphology and SEI through electrochemical processes.^19,20^ Generally, highly concentrated electrolytes [≥4 mol/L (M)]^21−24^ and functional additives or alternative salts/solvents^25−28^ are employed to induce compact Li metal plating through the formation of a stable SEI with good Li-ion conductivity. However, the large variety of systems studied make it difficult to establish a coherent perception on how the Li metal microstructural evolution and SEI composition/structure interact and how these impact the reversibility upon cycling. An opportunity to gain comprehensive understanding is variation of the salt concentration, being one of the very basic parameters that can be used to modify the electrolyte, determining the Li-ion mass transport through the electrolyte and through its solvation characteristics, also influence the SEI composition and structure, both of which play important roles in the evolution of Li metal microstructure.

In this work, we embark on a systematic study of the relationship between Li metal microstructure and the SEI composition driven from electrolytes with varying concentrations. Different concentrations of lithium bis(fluorosulfonyl)imide (LiFSI) salt were dissolved in a 1,2-dimethoxyethane (DME) solvent, where the high donor number makes DME effective in the dissociation of alkali metal salts, enabling the study of a wide salt concentration range in the same system. This allows us to elucidate the influence of other electrolyte properties on the microstructure of electrodeposited Li metal to explore the advantages and disadvantages of lower and higher salt concentrations with respect to the resulting SEI and Li deposition morphology. These results indicate the importance of achieving a high Li deposition coverage, which can be achieved at lower salt concentrations, thereby marrying the advantages of low- and high-molarity salts toward higher reversibility for Li metal batteries.

## Results and Discussion

### Impact of Electrolyte Concentrations on the Li Metal Morphology

**Figure 1:**
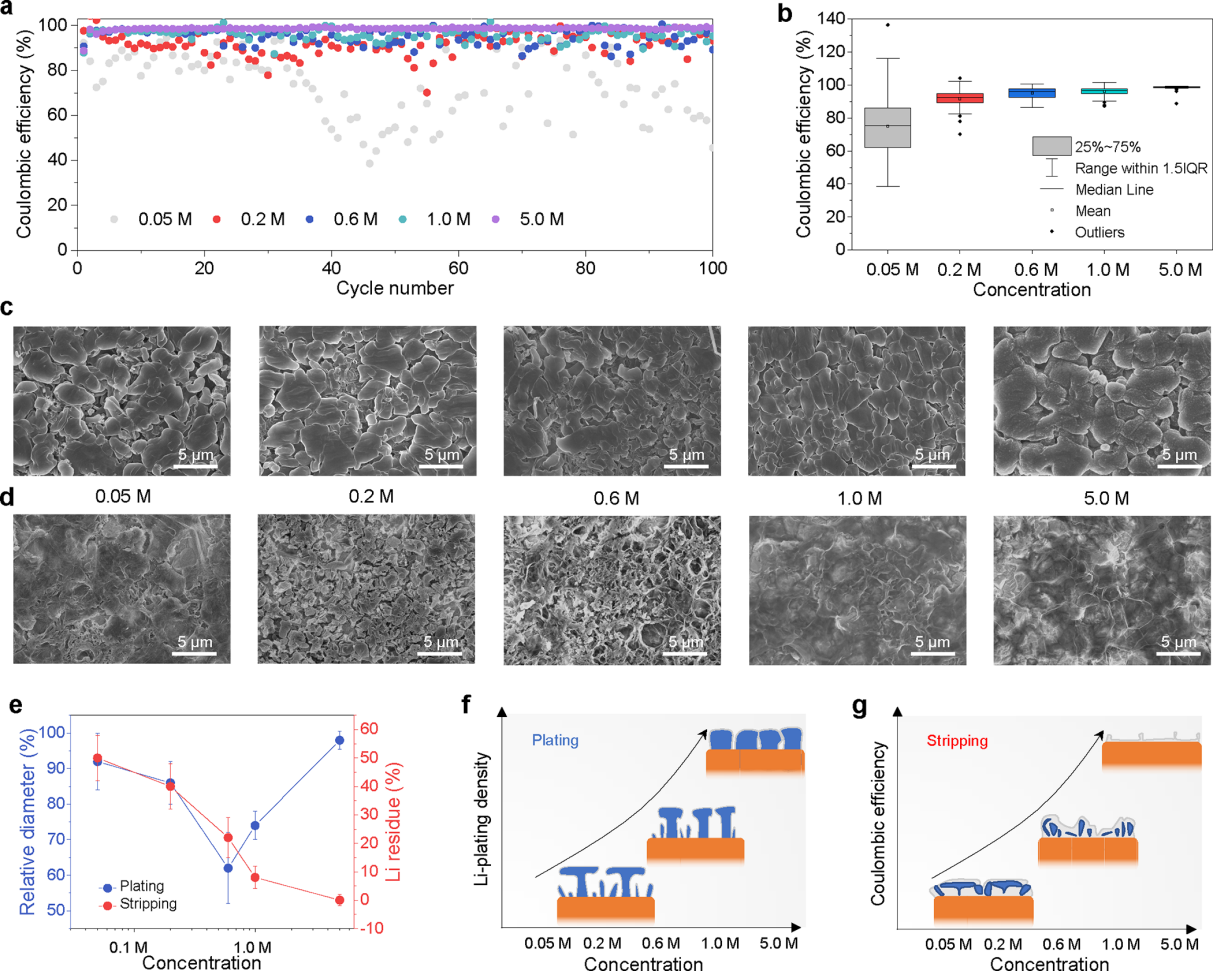
Electrochemical cycling and the Li metal microstructure. (a) CE in Li||Cu cells for LiFSI DME electrolyte with different molarities (0.05, 0.2, 0.6, 1.0, and 5.0 M). (b) Box plot obtained on the basis of (a) showing statistics of the CE. The center line of the box plot represents the median; lower and upper box limits represent the 25 and 75% quantiles, respectively; whiskers extend to box limit ±1.5 × IQR (interquartile range); outlying points plotted individually. (c) SEM images of Li deposited at 0.5 mA cm^–2^ for 2 h (1 mA h cm^–2^) after the plating under the different electrolyte concentrations. (d) SEM images of the Cu substrate after cycles, ending with Li stripping to 1.0 V vs Li/Li^+^ under the different electrolyte concentrations. The scale bars in (c,d) are 5 μm. (e) Estimation of the average diameter of Li metal deposits determined by SEM after plating, obtained from the images shown in (c,d). The relative diameter is normalized by the largest average particle size among five different electrolytes, and the Li residual is estimated by the surface coverage. (f,g) Schematic evolution of the Li metal morphology in Li||Cu cells as a function of concentration based on the SEM images for (f) discharge and (g) charge.

The reversibility of the Li metal plating/stripping in LiFSI DME electrolytes with different molarities is evaluated in Li||Cu cells. During the first 100 cycles at a current density of 0.5 mA cm^–2^ for 1.0 mA h cm^–2^, the average Coulombic efficiency (CE) increases with the salt concentration (Figures 1a and S1), along with a decrease in CE fluctuation (Figure 1b). A similar overall trend is observed for higher current densities of 1.0, 3.0, and 5.0 mA cm^–2^ for 1.0 mA h cm^–2^ (Figures S2–S5). The stability and overpotential are also evaluated in Li||Li symmetric cells (Figures S6 and S7) where the reduced overpotential with increasing molarity appears to be a consequence of the reduced interfacial charge-transfer resistance after the formation of SEI (Figure S8). The Li-ion transference number () of the electrolytes is obtained via the method of Abraham et al.,^29^ resulting in the largest value for the 1.0 M salt electrolyte (Figures S9–S13 and Table S1). A larger  is considered favorable as it extends Sand’s time, that is, the time until the Li ions in the electrolyte located near the surface of the Li metal are depleted, which is associated with the initiation of dendrite growth.^3,9^

Scanning electron microscopy (SEM) was used to study the morphology of the plated/stripped Li deposits on Cu in Li||Cu cells after plating at a current density of 0.5 mA cm^–2^ to an areal capacity of 1.0 mA h cm^–2^ (Figure 1c) and after stripping to 1.0 V vs Li/Li^+^ (Figure 1d). From the top-view SEM images, it is observed that the Li deposits in the dilute electrolytes, 0.05 and 0.2 M, exhibit a similar diameter compared to that in the concentrated 5.0 M electrolyte. Moreover, with increasing molarity, the average diameter of Li deposits first appears to decrease until 0.6 M and then increase, while it increases to 5.0 M, as shown in Figure 1e. To get a full understanding of their morphology, SEM on cross-sections was carried out so that the morphology as a function of depth can be investigated (Figure S14). For the dilute electrolytes (0.05 and 0.2 M), the deposited Li metal is more porous with smaller, whisker-like Li deposits near the current collector, whereas more compact deposition present at the top results in mushroom-like structures, which explains the larger relative diameter observed from the top-view SEM images. In contrast, when the molarity increases, larger columnar deposits form, leading to the larger diameter of the Li deposits. Therefore, a similar diameter in the top-view images of Li metal deposits in dilute and highly concentrated electrolytes represents a different growth mechanism and morphology as illustrated in Figure 1f. To investigate the morphology after stripping, SEM images after charging the Li||Cu cells were collected (Figure 1d). As the concentration increases, less Li residues can be observed on the Cu surface (Figure 1e), which is schematically shown in Figure 1g. The columnar Li deposits that occur at higher concentrations tend to decrease the formation of Li residual, which is favorable for the high reversibility; however, the gaps between the columnar deposits are found to limit the deposit density.

### Microstructure Evolution and Li Species Quantification

**Figure 2:**
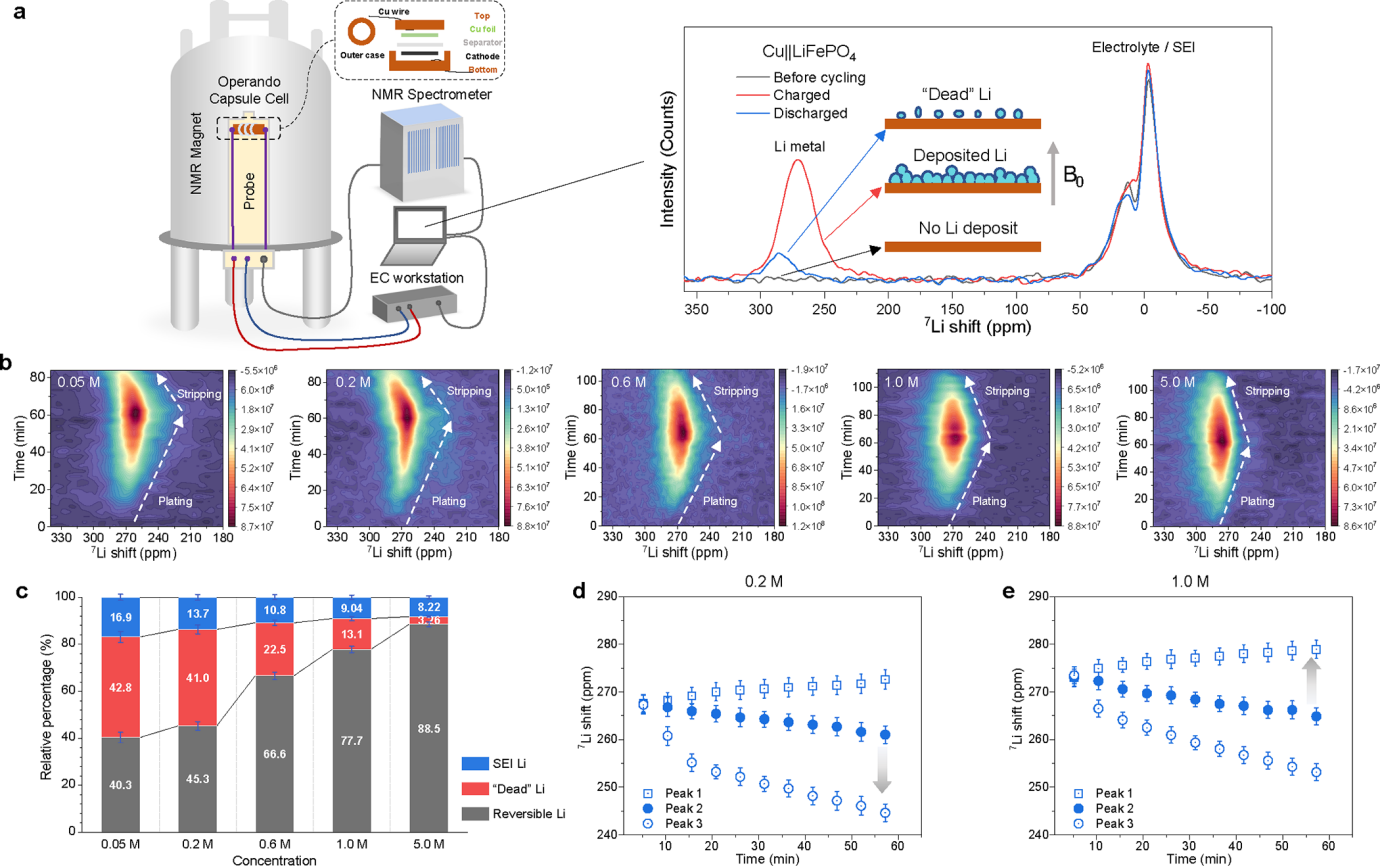
Operando ^7^Li NMR for quantification and microstructure evolution. (a) Schematic of the operando NMR setup. The dashed box shows the operando capsule cell inserted in the NMR probe coil. The cell is connected to an electrochemical workstation for galvanostatic charging/discharging. The figure on the right panel shows the spectra at different charge/discharge states, showing the resonance of the different Li species, including the Li metal, SEI (after its formation), and Li species in the electrolyte. The intensity of Li metal resonance at fully charged and discharged states is linked to the amount of deposited Li metal and “dead” Li, respectively. The gray arrow indicates the magnetic field, *B*_0_. (b) Operando ^7^Li NMR measurement during the first cycle of the Cu||LiFePO_4_ cells with different electrolyte concentrations at a current density of 1.0 mA cm^–2^. (c) Quantifying Li species after the first cycle, Li species in the SEI (blue bars), reversible Li metal (gray bars), and “dead” Li metal residual (red bars) species are derived from the Li metal integrated intensity ratio *I*(stripping)/*I*(plating) and the CE (Supporting Information Note 1). (d,e) Evolution of the Li metal resonances during charging in (d) 0.2 M LiFSI DME and (e) 1.0 M LiFSI DME electrolytes. Peak 1 and peak 3 stand for shoulder peaks on the left and right of peak 2, respectively (Figure S18). The gray arrow indicates the larger shift between the shoulder peak and the main peak.

^7^Li solid-state nuclear magnetic resonance (NMR) as a non-invasive method can provide quantitative and temporal information on Li metal deposition, where the development of operando probes allows us to monitor the processes of Li plating/stripping during an electrochemical measurement by recording spectra at intervals.^30−35^ The chemical shifts in ^7^Li solid-state NMR can be used to differentiate the metallic Li and diamagnetic Li species in the electrolyte and SEI (∼0 ppm), as well as provide insights into the evolution of the Li metal microstructure during cycling (Figure 2a). Here, ^7^Li operando NMR measurements are performed using an anode-less battery configuration of Cu||LiFePO_4_ cells.^36^ This plating and stripping process is shown in Figure 2b; upon charging the Cu||LiFePO_4_ cell, the Li metal resonance (∼272 ppm) grows, reflecting the Li metal deposition on the Cu current collector, and as expected, it subsequently shrinks upon Li stripping during discharge. The pristine ^7^Li spectra before charge, at the end of the first charge, and after subsequent discharge extracted from the operando dataset are shown for each electrolyte concentration in Figures S15 and 2a, which can be used for quantification. The Li metal resonance in the spectra after charging shows highest intensity compared to the other state of charge, indicating the total Li metal plated on the current collector. At the end of discharge, the Li metal resonance decreased compared to the charged state but still visible compared to pristine spectra, which can be related to the amount of “dead” Li. This is also shown in their differential spectra that as the molarity of the Li salt in the electrolyte increases from 0.05 to 5.0 M, the amount of “dead” Li decreases (Figure S16). Based on the NMR spectra and the CE, the amount of reversible Li metal, “dead” Li, and Li in the SEI species can be quantified with the method in Supporting Information Note 1, and the results are shown in Figure 2c and Table S2. The percentage of Li in the SEI and the “dead” Li both decreases with higher molarity, which consequently increases the capacity of the reversible Li metal. Only at 5.0 M, the capacity loss is not dominated by the “dead” Li metal, suggesting favorable SEI properties for highly concentrated electrolytes.

The evolution of the ^7^Li NMR resonance during charge and discharge can also provide insights into the evolution of the Li metal microstructure because the shift of the ^7^Li metal peak is sensitive to the orientation and microstructure of the Li deposits due to the bulk magnetic susceptibility.^31−33^ Generally, a pure Li metal strip gives rise to a resonance signal at ∼246 ppm when placed perpendicular to the fixed magnetic field *B*_0_, which shifts to higher ppm values when the strip is parallel to *B*_0_.^31,37^ Therefore, mossy or whisker-like microstructures growing perpendicular to Cu, assuming the electrodes to be perpendicular to *B*_0_, have been associated with a chemical shift range centered at around 272 ppm, whereas mossy microstructures encompass a broader spectral region covering a chemical shift range of approximately 250–290 ppm, and more compact Li metal appears to approach ∼246 ppm (Li metal strip).^33^ Also, the vicinity of the deposits to the current collector and the electrode, in combination with their bulk susceptibility, impacts the shift. Electrodeposits near the Li metal substrate result in a shift of ∼260 ppm, whereas whisker-like structures that extend further away from the surface appear at ∼272 ppm. Due to the small diamagnetism of Cu, the Li metal shift is hardly affected; however, the paramagnetic LiFePO_4_ can induce a +15 ppm shift approximately.^36^ As shown in Figure 2b, a distinct difference in distribution of chemical shifts is observed, broadening to smaller ppm values in low-concentration electrolytes while broadening to high ppm values in high-concentration electrolytes (Figure S17). These distributions can be deconvoluted in three resonances, where peak 1 and peak 3 stand for shoulder peaks on the left (higher ppm) and right (lower ppm) of the peak 2, respectively (Figures 2d,e and S18). In the 0.2 M electrolyte, peak 3 shifts significantly to lower ppm values along with peak 2 (Figure 2d), suggesting the formation of relatively compact horizontal Li metal microstructures at the end of plating, in line with the mushroom-like Li metal morphology observed with SEM (Figures 1f and S14). In the 1.0 M electrolyte, peak 2 and peak 3 also shift to smaller ppm values (Figure 2e), but the shift is relatively small compared to that for the 0.2 M electrolyte. However, peak 1 shows a greater shift to higher ppm values compared to peak 3. This indicates that gradually more compact deposition of perpendicular Li metal microstructure occurs, in line with the more columnar Li metal observed with SEM for this electrolyte concentration. The above results demonstrate that lower concentration (0.05–0.6 M) and higher concentration (1.0–5.0 M) LiFSI DME electrolytes result in different Li metal morphologies, which has profound impact on the evolution of the CE, “dead” Li, and SEI. In addition, the ^7^Li chemical shift at the very onset of Li plating tends to increase with increasing electrolyte molarity as shown in Figure S17, which at this early stage is difficult to explain by a microstructural effect. A possible explanation is a difference in the coverage of the Li deposits on the Cu current collector,^29,36^ that is, how much of the Cu current collector is covered by the Li metal, suggesting that the coverage decreases with increasing electrolyte molarity. To gain insights into the role of the early-stage nucleation and coverage, two electrolyte concentrations, 0.2 M representing a lower concentration and 1.0 M representing an intermediate concentration, are studied in more detail. The highly concentrated electrolyte is not selected because of its higher viscosity and cost, which makes it less attractive for future practical application.

### Li Nucleation and Initial Growth

**Figure 3:**
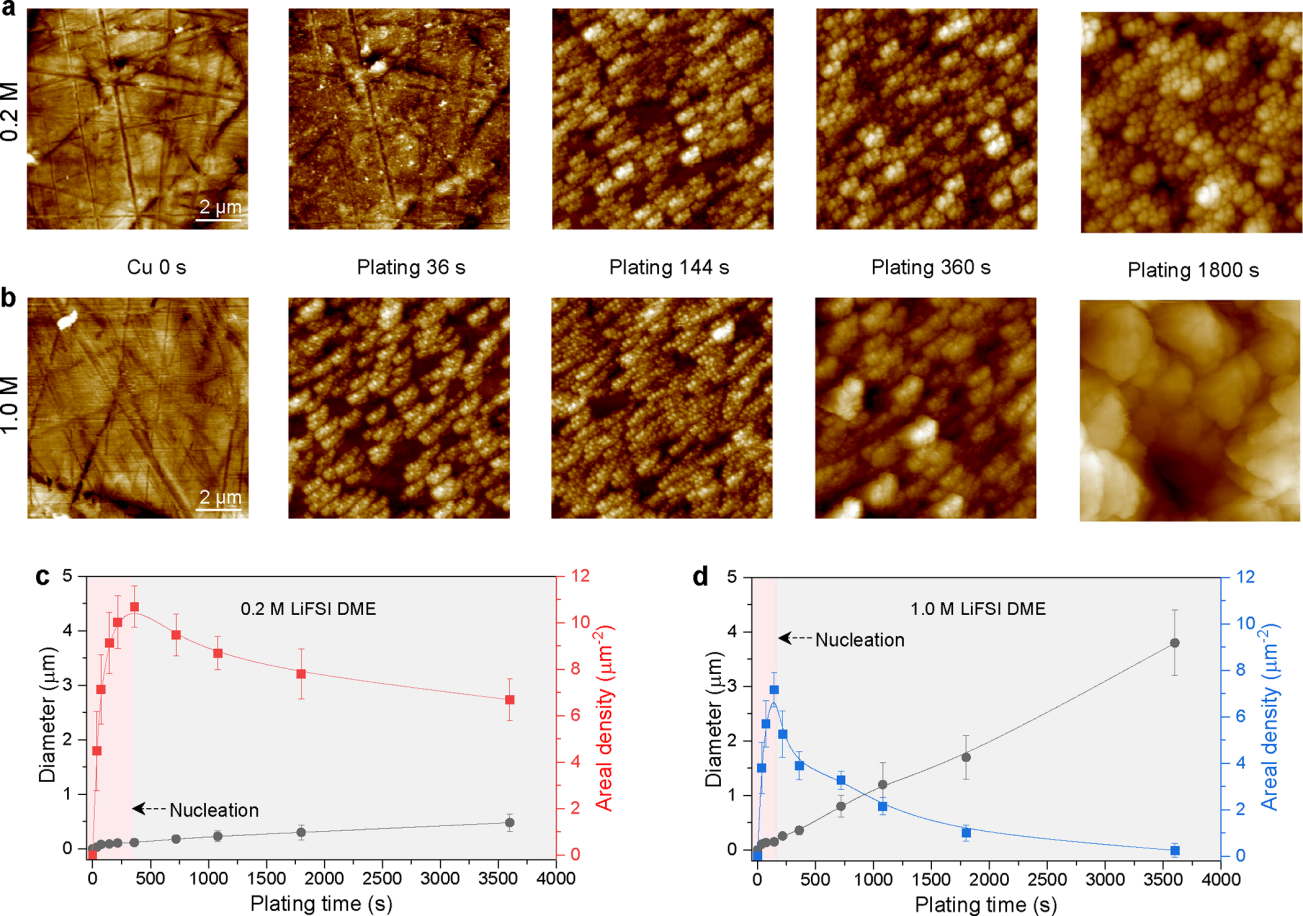
Li metal nucleation and growth from in situ AFM. (a,b) Topography of the Cu substrate before and after Li plating at 0.5 mA cm^–2^ for 36 s (0.005 mA h cm^–2^), 144 s (0.02 mA h cm^–2^), 360 s (0.05 mA h cm^–2^), and 1800 s (0.25 mA h cm^–2^) in (a) 0.2 M LiFSI DME and (b) 1.0 M LiFSI DME electrolytes using in situ electrochemical AFM measured on an area of 10 × 10 μm. Scale bar, 2 μm. (c,d) Diameter of Li metal deposits and the areal density evolution in (c) 0.2 M LiFSI DME and (d) 1.0 M LiFSI DME estimated from the AFM images, where the pink zone represents the nucleation process and the gray zone represents the following growth process.

To gain more insights into the nucleation and growth process, the initial Li deposition coverage and size of the Li metal deposits on the Cu substrate are investigated by in situ electrochemical atomic force microscopy (AFM). During these measurements, there is no applied pressure due to the nature of the in situ AFM setup (Figure S19) and thus represents different conditions from the morphologies shown in the SEM measurements (Figure 1c,d), where the pressure of the separator on the Li morphology can be expected to result in more compact plating. Figure 3a,b shows the AFM images before and after increasing the deposition time in the 0.2 and 1.0 M LiFSI DME electrolytes. Before Li deposition (0 s deposition time in Figure 3a,b), the grooves in the Cu surface present due to polishing are clearly resolved. After 36 s (0.005 mA h cm^–2^), a thin layer of nanosized Li deposits can be observed for 0.2 M (Figure 3a). In contrast, in 1.0 M electrolyte, the coverage of the Cu substrate is around half of the detect area (Figure 3b) with larger deposits of ∼100 nm in diameter. During subsequent deposition to 360 s (0.05 mA h cm^–2^), the number of Li metal deposits in the 0.2 M electrolyte increases continuously (Figure 3c). However, upon subsequent plating, the size of the Li metal deposits on top of this layer only marginally increases to 300–500 nm after 3600 s (0.5 mA h cm^–2^, Figure S20), and its number remains high as summarized in Figure 3c. In contrast, in the 1.0 M electrolyte, the number of Li metal deposits decreases sharply after 144 s (0.02 mA h cm^–2^), while the average size increases steadily (Figure 3d), approaching several micrometers in diameter at 1800 s (0.25 mA h cm^–2^) until a final diameter of around 4 μm is achieved (Figure S21). The decrease in the number of Li metal deposits appears to be a result of coalescence of smaller deposits, further supported by the cross-sectional SEM images shown in Figure S14. Based on the observation above, the increased coverage observed for the 0.2 M compared to that for the 1.0 M electrolyte therefore can be related to the lower shifts in 0.2 M than the 1.0 M electrolyte as observed in ^7^Li NMR spectra at the initial stage of plating (Figure 2b). A higher coverage of Li metal deposits, as observed for 0.2 M, can be considered favorable for dense Li metal growth. Although the larger cylindrical deposits at 1.0 M resulted in denser plating as can be seen from the SEM study, some pores were left behind such that the Cu substrate was not fully covered. On the other hand, the continuous nucleation of Li metal deposits in a longer time scale as observed for the 0.2 M electrolyte can be expected to lead to a higher final Li metal coverage and thus a denser Li metal film. However, this does not occur because the growth of the Li deposits is stalled in this dilute 0.2 M electrolyte to the final stages of deposition as indicated by operando ^7^Li NMR results. While the depletion-driven overpotential can qualitatively explain some of these aspects, another decisive factor in the growth of Li deposits is the SEI formation, which is significantly influenced by the electrolyte concentration.^38^

### SEI Structure and Composition

**Figure 4:**
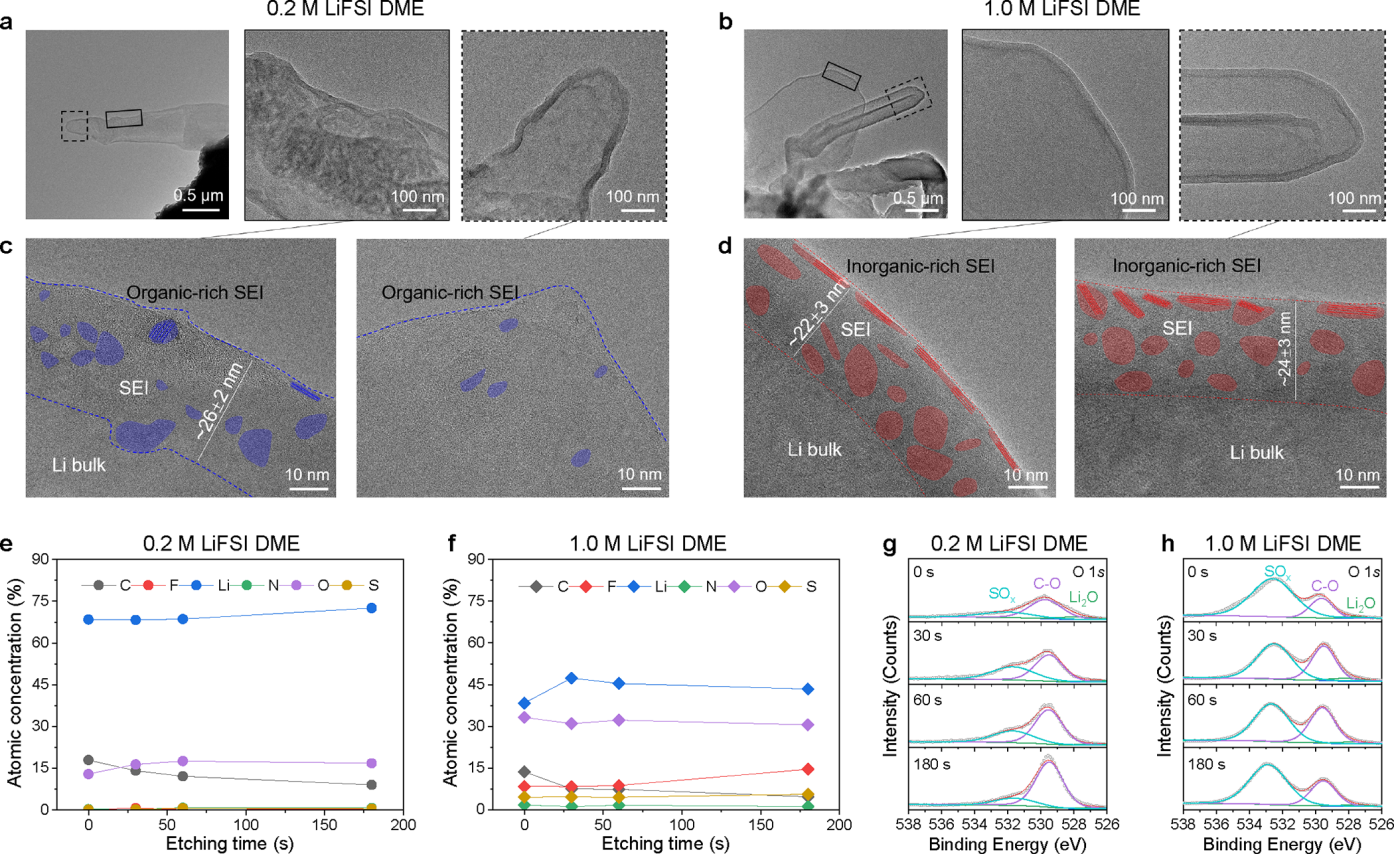
Structural and chemical analysis of SEI from cryo-TEM and XPS. (a) Bright-field cryo-transmission electron microscopy (cryo-TEM) image showing the morphology of Li metal deposits using the 0.2 M LiFSI DME electrolyte at a low magnification (left). The zoomed-in image of the region is marked by the black solid box (middle) and black dashed box (right). (b) Bright-field cryo-TEM image showing the morphology of Li metal deposits using the 1.0 M LiFSI DME electrolyte at a low magnification (left). The zoomed-in image of the region is marked by the black solid box (middle) and black dashed box (right). (c) High-resolution cryo-TEM images of the SEI layer on the deposited Li metal in a 0.2 M LiFSI DME electrolyte corresponding to (a) where the blue-colored area represents the inorganic components in SEI. (d) High-resolution cryo-TEM images of the SEI layer on the deposited Li metal in a 1.0 M LiFSI DME electrolyte corresponding to (b) where the red-colored area represents the inorganic components in the SEI. (e,f) XPS depth profiles after cycling for both 0.2 and 1.0 M LiFSI DME electrolytes (Li||Cu cells, 20 cycles at 0.5 mA cm^–2^ for 1.0 mA h cm^–2^) showing the SEI composition after different sputtering times on the deposited Li metal in (e) 0.2 M LiFSI DME and (f) 1.0 M LiFSI DME. (g,h) Deconvoluted O 1s XPS depth profiles as a function of time of the SEI formed in (g) 0.2 M LiFSI DME and (h) 1.0 M LiFSI DME.

Stabilized under the cryogenic conditions in TEM, the Li deposits and SEI structure are investigated with the 0.2 and 1.0 M LiFSI DME electrolytes, and the results are shown in Figure 4a–d. In the low-magnification cryo-TEM images of the Li metal plated in 0.2 M LiFSI DME (Figure 4a), whisker-like Li deposits are covered with an uneven SEI, resulting in a rougher surface (indicated by the light–dark variations in the SEI coating). Two areas were selected for higher magnification, marked with the solid box and dashed box. The thickness of the SEI for the 0.2 M electrolyte varies significantly. The SEI region of Figure 4a (left) is measured to be approximately 26 ± 2 nm, as shown by the high-resolution cryo-TEM images, where the interface between the deposited Li metal and the SEI is not well defined and irregular in shape. The SEI layer is dominated by amorphous components in which a small number of crystalline domains are randomly dispersed, forming a nanostructured mosaic SEI morphology.^39^ Most likely, the amorphous matrix represents organic species formed by DME solvent decomposition, whereas the crystalline grains represent the inorganic components of the SEI layer (Figures 4c and S22). The latter are attributed to Li_2_O and LiF from selected area electron diffraction (SEAD) measurements (Figure S23). From cryo-scanning TEM annular dark-field (ADF) images combined with electron energy loss spectroscopy (EELS) mapping, it can be concluded that the SEI formed in the 0.2 M electrolyte is mainly organic, being rich in carbon and oxygen (Figure S24). For the 1.0 M LiFSI DME electrolyte, the low-magnification cryo-TEM image in Figure 4b shows that the Li metal deposits have a larger diameter and the SEI is smoother and conformally covers the Li metal deposits. In this case, the SEI thickness is quite well defined with a thickness of around 22–24 nm, exhibiting a multilayer nanostructure.^40^ In the outer layer, the well-defined lattice fringes represent large crystalline grains (∼10 nm), whereas the inner layer is largely amorphous (Figures 4d and S22). The inorganic components in the outer layer are Li_2_O and LiF, as determined by SEAD (Figure S25). In the ADF and EELS mapping, a strong oxygen signal is present (Figure S26), further confirmed by X-ray photoelectron spectroscopy (XPS) measurements (Figure 4f).

Since the organic components of the SEI are mainly decomposition products of the DME solvent, the carbon content in the SEI is expected to be higher. This appears to be true for the organic-rich SEI (more organic components such as C–O and C–C/C–H species by solvent decomposition) formed in the 0.2 M electrolyte based on the XPS measurements in Figure 4e. In contrast, an inorganic-rich SEI (more inorganic components by anion decomposition) containing Li_2_O and LiF is expected to have a higher ratio of oxygen and fluorine. This appears to apply to the SEI formed in the 1.0 M electrolyte based on the XPS measurements in Figure 4f. The deconvoluted XPS depth profiles provide more detailed information on the impact of the electrolyte concentration on the SEI formation. In the 0.2 M electrolyte, the presence of a small fraction of SO_*x*_ and Li_2_O indicates very limited salt decomposition, consistent with the low intensity of F-, S-, N-containing compounds (Figures S27–S31). The large fraction of C-containing species detected in the SEI formed in the 0.2 M electrolyte indicates that solvent decomposition dominates the SEI formation (Figures 4g and S32), which is consistent with the large redox peak observed at ∼0.5 V using cyclic voltammetry (Figure S33). The higher salt concentration in the 1.0 M electrolyte results in more SO_*x*_ and Li_2_O, suggesting that LiFSI decomposition dominates the SEI formation process (Figure 4h), which is consistent with the higher LiF intensity in the Li 1s spectrum and the lower fraction of carbonate species (Figures S31 and S32).

### Joining the Advantages of Lower and Higher Concentration Electrolytes

The higher nucleation coverage and longer nucleation periods in low-concentration electrolytes, driven by the more severe Li-ion depletion, can be considered as a favorable starting point for dense Li metal plating and thus for more reversible cycling. However, the same ion depletion is responsible for a more organic-rich SEI through DME solvent decomposition, which promotes inhomogeneous Li plating/stripping and stalls the growth of large and dense Li metal deposits. The larger surface area of the smaller deposits in dilute electrolytes causes more SEI growth, which leads to more irreversible capacity loss and electrolyte consumption during cycling. The benefit of a higher salt concentration is the thin, well-defined multi-layer SEI that is rich in inorganic species that guarantee a higher stability as well as a higher and more homogeneous Li-ion conductivity. This seems to be responsible for the continuous growth of large and dense Li metal deposits, which in turn suppresses the formation of “dead” Li metal, and the smaller surface area of these large deposits leads to a much smaller amount of SEI species, both these factors promoting the reversibility. The disadvantages are however that the lower nucleation density at higher electrolyte concentrations leaves parts of the Cu uncovered, leading to a lower Li deposition coverage and limiting the Li metal density. This in addition to the known disadvantages of higher salt concentrations, that is, the increase in viscosity (lowering conductivity), reduction in wettability, and increase in costs, has so far limited its practical application.^41^

**Figure 5:**
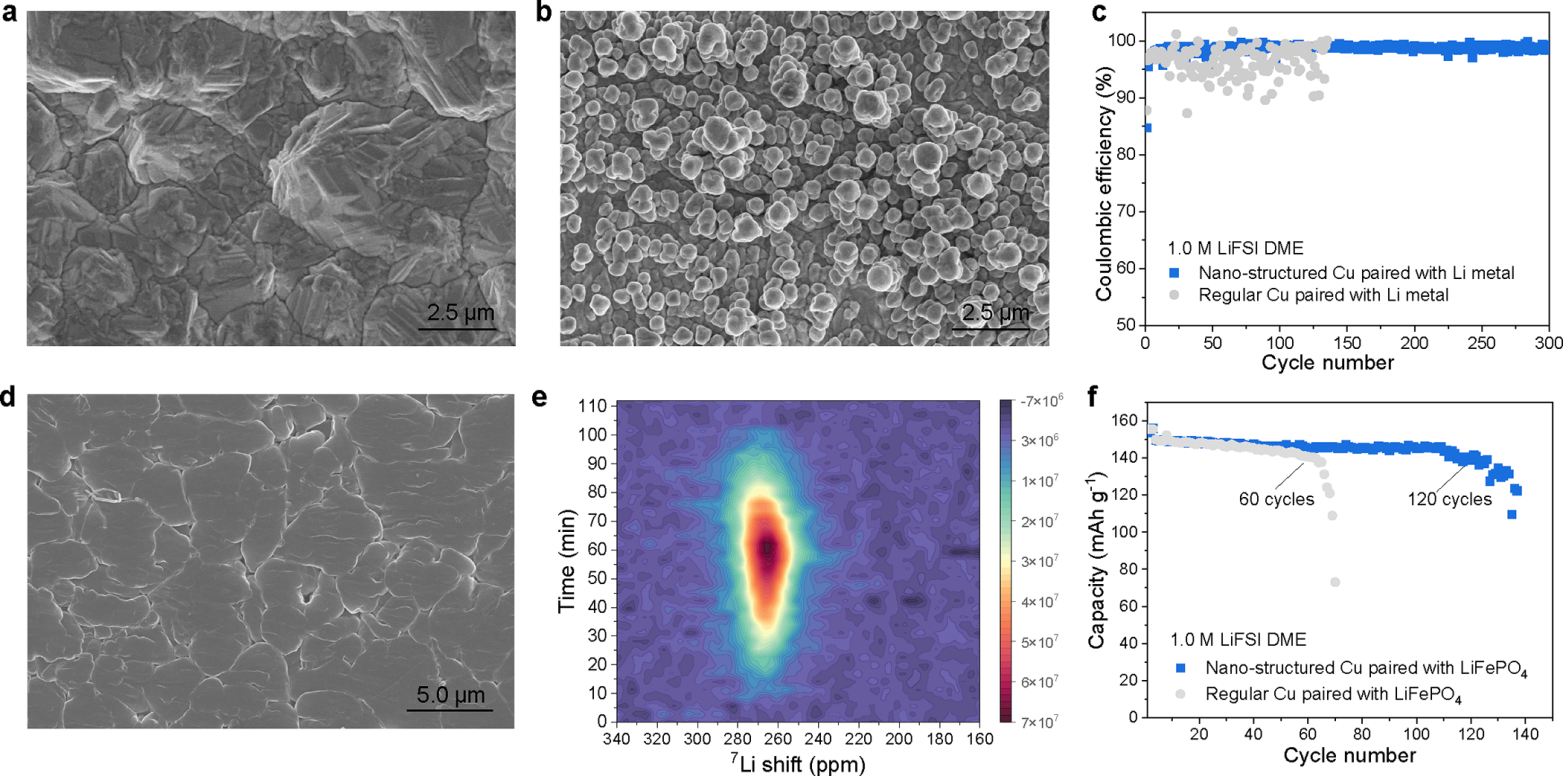
Increasing the Li deposition coverage for the 1.0 M LiFSI DME electrolyte. (a,b) Top-view SEM image of the (a) regular Cu foil and (b) nanostructured Cu foil. (c) CE from Li||Cu cells using the regular and nanostructured Cu in a 1.0 M LiFSI DME electrolyte cycled at a current density of 0.5 mA cm^–2^ to a capacity of 1.0 mA h cm^–2^. (d) Top-view SEM image of the deposited Li metal on the nanostructured Cu in a 1.0 M LiFSI DME electrolyte cycled at a current density of 0.5 mA cm^–2^ to a capacity of 1.0 mA h cm^–2^. (e) Operando ^7^Li NMR spectra acquired during the first cycle of Cu||LiFePO_4_ cells using the nanostructured Cu in a 1.0 M LiFSI DME electrolyte. (f) Capacity retention of Cu|| LiFePO_4_ batteries cycled at C/3 in a 1.0 M LiFSI DME electrolyte using different Cu foils with pre-deposit Li of 0.5 times the capacity of cathode electrodes. The areal capacity of the LiFePO_4_ cathode is 2.0 mA h cm^–2^.

Therefore, the challenge is to combine the favorable properties of both high and low salt concentrations. With respect to the SEI composition, a stable SEI requires at least an intermediate, around 1.0 M, salt concentration (excluding the possibility of improving the SEI with additives). Therefore, a rational strategy is to aim for increasing the density in nucleation sites at 1.0 M to achieve denser plating while keeping the favorable SEI morphology and composition. Several studies have demonstrated that initial nucleation pulses can increase the density of Li metal nucleation on the electrode surface.^42−44^ However, the pulsed charging/discharging induces continuous consumption of both solvent and Li from the cathode side, which leads to more rapid degradation of the battery.^45^ Instead, we propose to the commercially available current collector covered by nanosized Cu particles to replace the regular Cu current collector (Figure 5a) that acts as nucleation centers for Li metal growth (Figures 5b, S34, and S35) and study the deposition in combination with a 1.0 M LiFSI DME electrolyte.

Introducing the nanostructured Cu in a Li||Cu cell demonstrates a marked improvement in the average CE and in cycling stability as compared to the regular Cu (Figure 5c). The SEM images demonstrated that the nanostructured Cu results in more compact and smoother Li deposition as compared to the regular Cu (Figures 5d and S36). Operando ^7^Li NMR was performed to further verify the Li metal microstructural evolution on the nanostructured Cu. Compared with using regular Cu, the Li metal peak on nanostructured Cu results in a chemical shift at lower ppm values in the initial stages (Figures 5e and S37), resembling the chemical shift observed in dilute electrolytes, indicating an increased Li deposition coverage. The nucleation on this nanostructured Cu was further studied by in situ electrochemical AFM, where a larger nucleation size was achieved as expected for the 1.0 M electrolyte (Figure 3), but in this case in combination with a higher coverage (Figure S38). In order to evaluate the potential application, Cu||LiFePO_4_ full cells were assembled to study the cycling stability of the nanostructured and the regular Cu. The results show stable cycling over 130 cycles for the nanostructured Cu, and the cell using regular Cu fails after around 60 cycles (Figures 5f and S39), extending the cycling life more than two times.

### Demonstration of the Li Deposition Coverage in a Commercial Electrolyte

**Figure 6:**
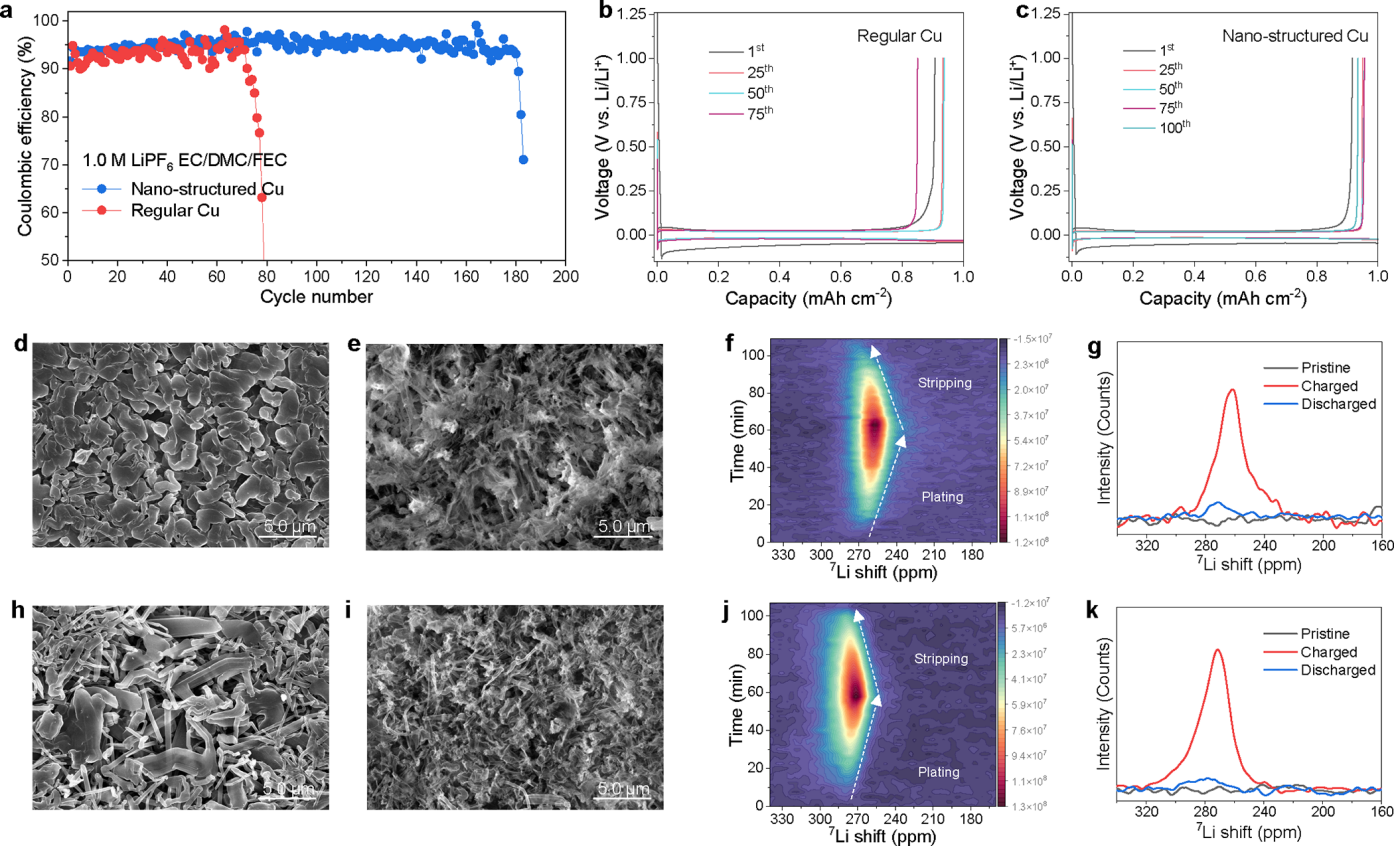
Increasing the Li deposition coverage in the ester electrolyte. (a) CE of Li||Cu cells using regular and nanostructured Cu, cycling at a current density of 0.5 mA cm^–2^ to a capacity of 1.0 mA h cm^–2^. (b,c) Corresponding charge/discharge curves of Li metal plating/stripping on (b) regular and (c) on the nanostructured Cu. (d,h) Top-view SEM image of the plated Li metal on (d) nanostructured and (h) on the regular Cu after three cycles and then plating at 0.5 mA cm^–2^ to a capacity of 1.0 mA h cm^–2^. (e,i) Top-view SEM images of (e) nanostructured and (i) regular Cu after Li stripping to 1.0 V vs Li/Li^+^. (f,j) Operando ^7^Li NMR spectra acquired during the first cycle of a Cu||LiFePO_4_ cell using the (f) nanostructured Cu and (j) regular Cu. (g,k) ^7^Li NMR spectra using the (g) nanostructured Cu and (k) regular Cu before (pristine), after Li plating (charged) and after Li stripping (discharged). The electrolyte for these cells is 1.0 M LiPF_6_ in EC/DMC (1:1 in weight) with 5% FEC.

Finally, the impact of increasing the Li deposition coverage and density via the nanostructure of the Cu substrate is investigated using a typical ester electrolyte. The standard carbonate electrolyte with 1.0 M LiPF_6_ dissolved in ethylene carbonate and dimethyl carbonate (EC/DMC 1:1 in weight), including a 5% FEC additive which is known to improve the cycling stability of Li metal. The electrochemical cycling of the Li||Cu cells demonstrates that also in this case, the nanostructured Cu results in a higher CE (91.6 vs 90.5%) and better cycling stability (Figure 6a), as well as a lower overpotential (Figure 6c), as compared to regular Cu under the same conditions (Figure 6b). The Li metal deposits on the nanostructured Cu have a larger diameter (Figure 6d) than those on the regular Cu, where the latter exhibits whisker-like microstructures (Figure 6h). After Li stripping, the regular Cu shows more whisker-like Li residuals left on the surface (Figure 6i), compared to that from the nanostructured Cu (Figure 6e). Operando ^7^Li NMR was carried out to gain insights into the Li metal microstructure evolution during plating and stripping in the ester electrolyte using the regular or nanostructured Cu in Cu||LiFePO_4_ cells. Comparing the nanostructured and regular Cu shown in Figure 6f,j, respectively, it can be seen that the nanostructured Cu leads to lower ^7^Li chemical shifts that can be associated with more compact plating and larger deposition coverage. In contrast, the deposition on regular Cu results in a higher shift for the ^7^Li chemical shifts, indicating more whisker-like growth and less deposition coverage (Figure 6g,k). Interestingly, based on the ^7^Li NMR spectra after discharge, there is little difference in the amount of residual lithium metal between the two copper current collectors, which may be related to a similar SEI composition, also suggested by the similar CE. Even though the improvement in the CE upon cycling is small, the more compact plating on the nanostructured Cu enhances the cycling stability, extending the cycle life of the Li||Cu cell.

### Comprehensive Picture of the Li Metal Microstructure

**Figure 7:**
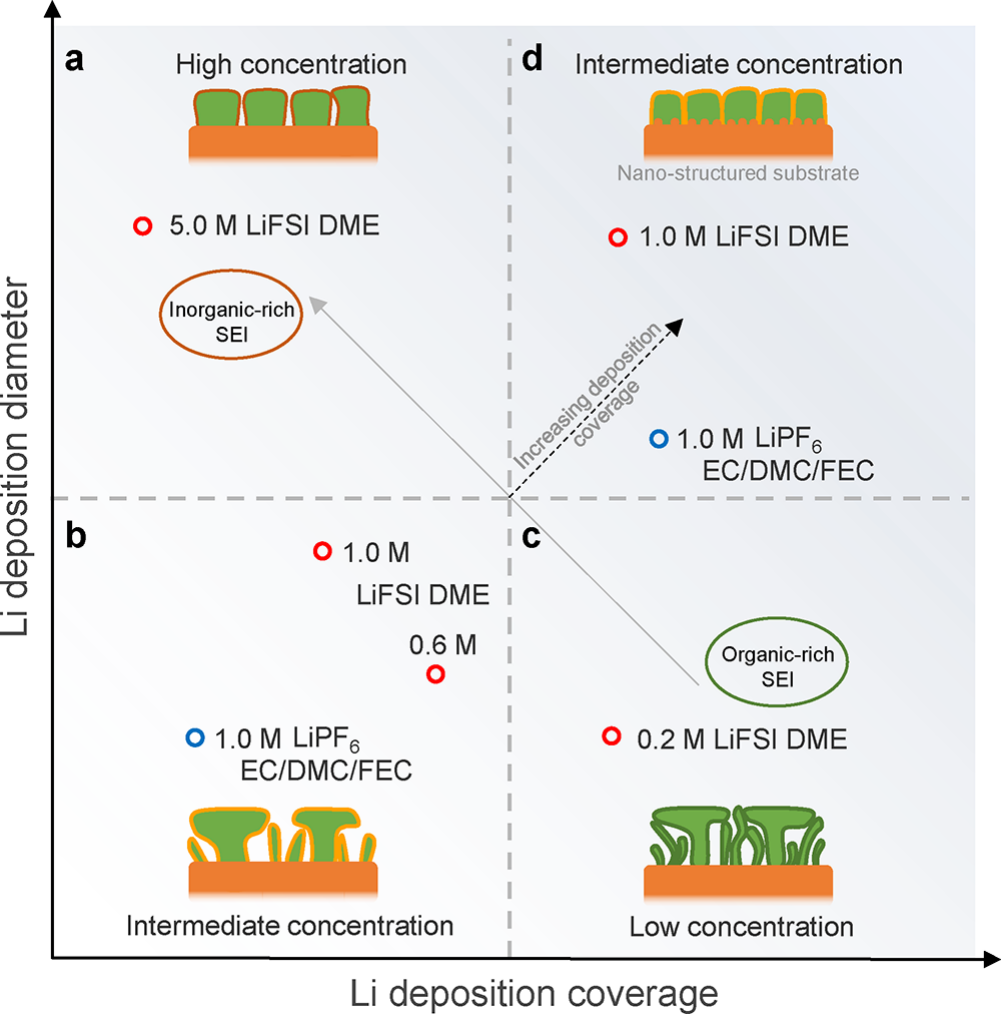
Correlation between the Li deposition coverage, Li deposition diameter, and electrolytes. Four regions of the Li metal morphology can be distinguished that clarify the relationship. (a) Highly concentrated electrolytes result in low deposition coverage and a large Li deposition diameter. (b) Intermediate concentration electrolytes result in small Li deposition coverage and a small Li deposition diameter. (c) Low-concentration electrolytes result in high deposition coverage and a small Li deposition diameter. (d) Intermediate concentration electrolytes result in high deposition coverage when introducing a substrate with a high density of nucleation sites, resulting in a large Li deposition diameter in combination with high coverage.

Based on the above observations, the complex dependence of the deposition morphology on the concentration due to the different nucleation conditions as well as the different SEI growth conditions can be clarified. To achieve dense plating, it not only requires large Li deposition diameters, as achieved in higher concentrated electrolytes, but also requires a high deposition coverage. This is demonstrated by Li plating in 5.0 M LiFSI DME, where large Li deposit diameters are realized, but the low deposition coverage can be held responsible for leaving gaps between the deposits (Figure 7a), which compromises the Li metal density. In lower concentration electrolytes, such as 0.2 M LiFSI DME, the deposition coverage is high, but it results in a mosaic-structured organic-rich SEI, which does not support homogeneous plating or stripping of the Li metal (Figure 7c) and consequentially results in porous Li metal deposition and low reversibility. The favorable properties of both extremes can be combined in electrolytes with intermediate concentrations, such as 0.6 and 1.0 M, inducing a higher Li deposition coverage via the current collector surface structure (moving from Figure 7b–d). The investigation of the Li metal morphology and SEI structure as a function of electrolyte concentration demonstrates the importance of achieving a high Li deposition coverage, in combination with the conditions to grow large deposits, promoting denser Li metal deposition, a prerequisite to reversible Li metal batteries.

## Conclusions

In summary, the Li microstructure was systematically investigated as a function of electrolyte concentration using a combination of operando, in situ and ex situ experimental techniques that probe the Li metal morphology and SEI on all length scales, formulating a comprehensive picture of the relationship between the Li deposition coverage and microstructure in lithium metal batteries. The higher deposition coverage can be formed in the dilute electrolytes, which provide a favorable starting point for dense Li metal deposition. However, the formation of the organic-rich mosaic SEI, also a consequence of salt depletion at the Li metal surface, prevents the growth of large Li deposits and dense Li metal deposition. In contrast, higher concentrated electrolytes induce a thin and stable SEI, which induces the growth of large Li deposits. In this case, however, the relatively small deposition coverage limits the final density of the Li metal deposition. These results imply the importance of deposition coverage in the microstructure of Li metal. Furthermore, the deposition coverage can be improved through the substrate surface structure, making it possible to combine the favorable aspects of low-concentration electrolytes with those of highly concentrated electrolytes. For intermediate concentration electrolytes, the combination of the high deposition coverage with stable SEI driven from the functional additives or alternative salts/solvents provides a promising research direction for practical applications, which has also been demonstrated by the commercial carbonate electrolytes.

## Supporting Information Available

Detailed materials, experimental procedures, characterization methods, supporting notes, and figures including electrochemical performance, EIS, SEM, NMR, AFM, cryo-TEM, XPS, and XRD characterizations, as well as properties of electrolytes (PDF)
