# Supplementary material for: Clarifying the Relationship between the Lithium Deposition Coverage and Microstructure in Lithium Metal Batteries
Source: J Am Chem Soc. 2022 Nov 23;144(48):21961–71. doi: 10.1021/jacs.2c08849 (PMC9732870; doi:10.1021/jacs.2c08849)
Supplement: Supplementary file 1 — ja2c08849_si_001.pdf [file ja2c08849_si_001.pdf]

Supporting information

Clarifying the relationship between lithium deposition coverage and microstructure in lithium metal batteries

Qidi Wang[1,†], Chenglong Zhao[1,†,*], Shuwei Wang[2,†], Jianlin Wang[3], Ming Liu[1], Swapna Ganapathy[1], Xuedong Bai[3], Baohua Li[2,*], Marnix Wagemaker[1,*]

[1]Department of Radiation Science and Technology, Delft University of Technology, Delft 2629JB, The Netherlands

[2]Shenzhen Key Laboratory on Power Battery Safety and Shenzhen Geim Graphene Center, School of Shenzhen International Graduate, Tsinghua University, Guangdong 518055, China

[3]State Key Laboratory for Surface Physics, Institute of Physics, Chinese Academy of Sciences, Beijing 100190, China

[†]Q.W., C.Z. and S.W. contributed equally to this work.

*Corresponding authors: c.zhao-1@tudelft.nl, libh@mail.sz.tsinghua.edu.cn, m.wagemaker@tudelft.nl.

Methods

Materials:

Lithium metal foils (thickness of 250 μm) and Cu foils were purchased from MTI Corporation. All lithium metal foils were washed 3 times with DME solvent before use. Cu foils were immersed in diluted acetic acid for 3 min, subsequently washed with deionized water and acetone three times, separately, then they were quickly dried in the vacuum chamber of glove box at room temperature. The nano-structured Cu was purchased from Mingyu metal company. Battery-grade Dimethoxyethane (DME) and 4-Fluoroethylene carbonate (FEC) was purchased from Sigma-Aldrich, which was dehydrated with a 4 Å molecular sieve (Sigma-Aldrich) to eliminate any trace water before use. Lithium bis(fluorosulfonyl)imide (LiFSI) and $LiPF_6$ was purchased from Sigma-Aldrich, which were dried at 100 °C under vacuum for 24h before use.1.0 M $LiPF_6$ in EC: DMC (1:1, by weight) electrolyte was purchased from Sigma-Aldrich. All the electrolytes were prepared and stored in an Ar-filled glove box (H_2O < 0.1 ppm, O_2 < 0.1 ppm). $LiFePO_4$ was obtained from Leneng Technology for which the cathodes were prepared by mixing $LiFePO_4$ material, poly (vinylidene difluoride) (PVDF, MTI) binder and Super P (Alfa Aesar) conductive carbon in a weight ratio of 92:4:4. The resulting slurry was cast on the Al foil then dried at 60 °C for 6 h, followed by drying overnight at 120 °C in a vacuum oven.

Electrochemical measurements:

For the electrochemical cycling tests, all batteries were assembled into CR2032 coin cells in an Ar-filled glove box (H_2O < 0.1 ppm, O_2 < 0.1 ppm) with Celgard 2500 separator. 70 μL electrolytes were injected into each coin cell for comparison. All coin cells were tested using multi-channel battery testing systems (Land CT2001A or Lanhe G340A) at room temperature. Symmetric Li||Li cells were assembled to study the cycling stability under different current densities with different electrolytes, 15.6 mm diameter lithium metal foils with a 250 μm thickness were used as both the working and counter electrodes. For Li||Cu cells, 14 mm diameter lithium metal foils were used as the reference, while 16 mm Cu foils was as a working electrode with the effective area for Li deposition of 1.54 cm^2. During cycles, capacity of 1 mAh cm^{-2} Li was deposited on Cu foils at various current densities and the cut-off voltage for stripping was set to 1.0 V vs. Li/Li^+.

The electrochemical cycling performance of Cu||$LiFePO_4$ cells were tested in galvanostatic mode within a voltage range of 2.5–3.8 V. The cathodes had a diameter of 12 mm and loading of 2 mAh cm^{-2}. The Cu current collector was used after pre-deposition Li metal to 0.5 times the capacity of cathode electrodes. Cyclic voltammetry (CV) of Li||Cu cells with various electrolytes was conducted at a scan rate of 0.8 mV s^{-1} from -0.1 to 2.5 V vs. Li/Li^+. Electrochemical impedance spectra (EIS) of the symmetric cells were collected on an Autolab (PGSTAT302N) in the frequency range of 0.1 Hz–1 MHz with a potential amplitude of 10 mV.

Lithium ion transference number (t_{Li^+}) of electrolytes was measured via the method of Abraham et al.[1] The polarization potential (ΔV) of 10 mV was used for symmetric Li||Li cells with various kinds of electrolytes until the polarization currents reached a steady state, and the corresponding EIS measurements were collected both before and after the polarization. The t_{Li^+} was calculated as following equation:

$$t_{Li^+} = \frac{I^{ss}R_b^{ss}(\Delta V - I^0 R_i^0)}{I^0 R_b^0(\Delta V - I^{ss} R_i^{ss})} \qquad \text{Equation S1}$$

where ΔV is the applied potential, I^0 is the initial current and I^{ss} is the steady-state current; R_b^0 and R_b^{ss} are the initial and steady-state values of the bulk resistances; R_i^0 and R_i^{ss} are the initial and steady-state values of the interfacial resistances, respectively, which were determined by impedance measurements before and after the potentiostatic polarization.

Ionic conductivity of electrolytes was measured using symmetric stainless steel||stainless steel cells by collecting electrochemical impedance (R) at room temperature, and calculated using the following equation:

$$\sigma = \frac{L}{R \times S} \qquad \text{Equation S2}$$

where σ is ionic conductivity, S is the effective area of electrode, L stands for the thickness between two stainless steel electrodes, respectively. Test cells were assembled with a Polytetrafluoroethylene (PTFE) ring between two stainless-steel electrodes. Hence, the effective area of electrode is calculated based on the inner diameter of the PTFE ring, and the thickness between two stainless steel electrodes is based on the thickness of the PTFE ring.

Materials characterization:

Morphologies of electrodes were measured on a scanning electron microscope (SEM, HITACH SU8010). The obtained SEM images were analyzed with the PC-SEM (Hitachi SU8000 series) analysis software, and the particle size and number are further confirmed by the public domain software for processing and analyzing scientific images of Image J. Elemental composition on the surface of electrodes was analyzed by X-ray photoelectron spectroscopy (XPS, PHI 5000 VersaProbe II) using monochromatic Al Kα X-ray source. A sputter argon ion gun was equipped for depth profiling of the electrodes. Peaks were fitted using MultiPak software calibrated with respect to carbon (284.8 eV). The above morphology and composition characterizations were performed with cells being disassembled after specific cycles in an Ar-filled glove box and rinsed with pure DME solvent three times to remove residual electrolyte, followed by drying in the glove box for several hours at room temperature to remove the residual solvent. Then electrodes were transferred into the vacuum transfer boxes to avoid air exposure. Powder X-ray diffraction (XRD) was performed using a Bruker D8 Advance diffractometer equipped with a Cu Kα radiation source (λ_1=1.54060 Å, λ_2=1.54439 Å at 40 kV and 40 mA) and a LynxEye_XE detector.

In-situ atomic force microscopy (AFM) characterization

In-situ electrochemical AFM measurements (Bruker Corp., Dimension Icon) were performed with a three-electrode cell powered by an electrochemical workstation (CHI760E). Cells were assembled in an argon-filled glove box (H_2O < 0.1 ppm, O_2 < 0.1 ppm) with Cu substrate as working electrode and Li strips (diameter of 1mm) as the counter and reference electrodes. Li was deposited at 0.5 mA cm^{-2}, and in-situ AFM observation was carried out under open-circuit conditions after a specific time of deposition. AFM topography images were collected with the peak force tapping mode and the ScanAsyst-Fluid tips (k = 0.7 N m^{-1}, Bruker Corporation) were used for their superior force control with a pN-level force between tip and electrode, diminishing the damage to sample surface in the liquid condition. The obtained AFM images were analyzed with the NanoScope Analysis software, and the particle size and number are further confirmed by Image J.

Cryo-transmission electron microscopy (Cryo-TEM) characterization

Cryo-(S)TEM experiments were performed on scanning transmission electron microscope (STEM) (JEM-ARM300F, JEOL Ltd.) operated at 300 kV with a cold field emission gun and double Cs

correctors. The microscope was equipped with Gatan OneView and K2 cameras for images recording. During image acquisition, the corresponding electron dose flux (units of number of electrons per square angström per second, e^{-} $Å^{-2}$ s^{-1}) was recorded. Cryo-TEM images were obtained with an exposure time for each image of around 0.3 s with built-in drift correction function in GMS3 using the OneView and K2 camera. Cryo-TEM images were taken with an electron dose rate of 50 - 500 e^{-} $Å^{-2}$ s^{-1}. Short-exposure single-frame shots were used to estimate the defocus and make it as close as possible to Scherzer defocus. The EELS spectrum images were recorded with a camera length of 20 mm, and a pixel dwell time of 10 ms. Energy drift during spectrum imaging was corrected by centering the zero-loss peak to 0 eV at each pixel. Elemental maps were computed through a two-window method in a pre-edge window fitted to a power-law background and a post-edge window of 50-200 eV on the core-loss signal. Analysis of the spectra has been performed in Digital Micrograph.

For cryo-TEM preparation of Li-metal anode, a lacey carbon TEM grid was put on Cu foil working electrode and assembled into Li‖Cu cells in an argon-filled glovebox. The cells were discharged at a constant current density of 1.0 mA cm^{-2} for 15 min, then TEM grid was taken out by disassembling the cells for measurement. TEM grid was carefully transferred into the cryo-TEM holder in glovebox with a specialized shutter to prevent air exposure and ice condensation onto the sample introducing any side reactions. Once the cryo-TEM holder was transferred into TEM column, the temperature was maintained at around -170 °C using liquid nitrogen. All cryo-TEM images were taken at around -170 °C to reduce beam damage.

Solid-state NMR characterization

Operando solid-state NMR measurements were conducted on a wide-bore Bruker Ascend 500 system equipped with a NEO console with a magnetic field strength of 11.7T and a ^{7}Li resonance frequency being 194.37 MHz using a solenoidal Ag-coated Cu coil. Operando static ^{7}Li NMR measurements were performed using an automatic-tuning-and-matching probe (ATM VT X WB operando NMR probe, NMR Service) at room temperature which can allow for an automatic recalibration of the NMR radio-frequency (rf) circuit during an operando electrochemistry experiment. A highly shielded wire with low-pass filters was attached to the probe for electrochemical measurement, which could minimize the interferences between NMR and the

electrochemistry circuit. Single-pulse with a π/2 pulse of 4 μs and recycle delay of 1.0 s was applied to acquire the 1D static spectrums. The electrochemical cell was simultaneously controlled by a Maccor battery testing system. A plastic capsule cell made out of polyether ether ketone (PEEK) was used for operando NMR experiments. The cells were assembled using $LiFePO_4$ cathode (areal capacity is 2.0 mAh cm^{-2}) and Cu foils as working and counter electrodes with both a piece of Celgard and a piece of Glass fiber (Whatman GF/A) as separator. Before measurements, the assembled cells were rested for 2 h in glovebox. The operando capsule cell was aligned in an Ag-coated Cu coil with $LiFePO_4$ and Cu foil electrode were oriented perpendicular to B_0 and parallel with respect to the B_1 rf-field. During the static ^{7}Li NMR measurements the cells were cycled at current density of 1.0 mA cm^{-2}. The charge cut-off capacity of 1 mAh cm^{-2} was used for Li-metal plating on Cu foils, and a discharged cut-off voltage is 2.0 V for Li stripping. During the charge and discharge process, NMR spectra were continuously acquired, each with a scan time of about three minutes. The chemical shift of ^{7}Li was referenced to 1.0 M aqueous solution of LiCl at 0 ppm. Bruker Topspin and Mestrenova software were used for data processing.

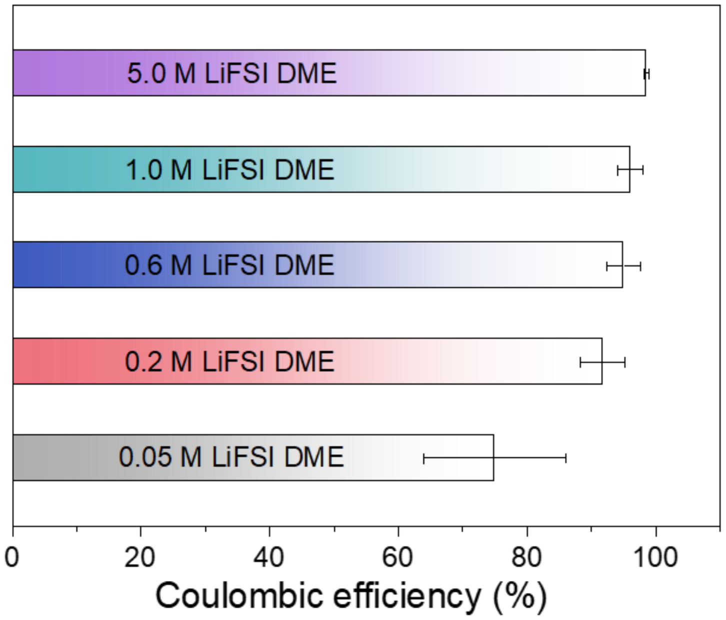

Figure S1. Average Coulombic efficiency (CE) of Li||Cu cells using different electrolytes for 100 cycles. The cells were cycled at the current density of 0.5 mA cm^{-2} to an areal capacity of 1 mAh cm^{-2}.

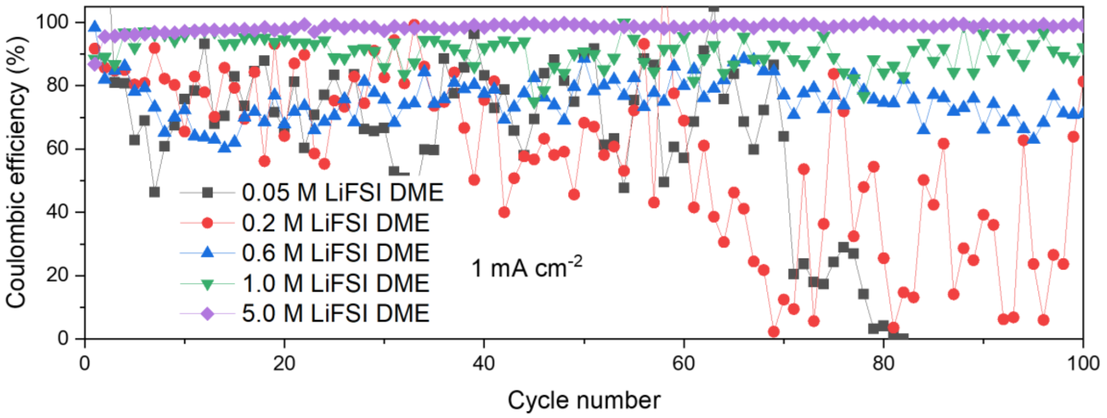

Figure S2. Cycling performance of Li||Cu cells with different electrolytes at a current density of 1 mA cm^{-2} to an areal capacity of 1 mAh cm^{-2}.

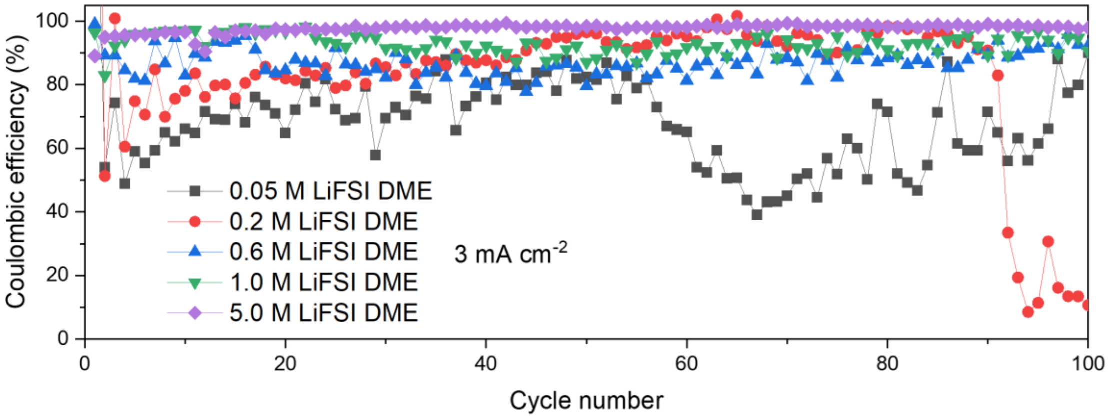

Figure S3. Cycling performance of Li||Cu cells with different electrolytes at a current density of 3 mA cm^{-2} to an areal capacity of 1 mAh cm^{-2}.

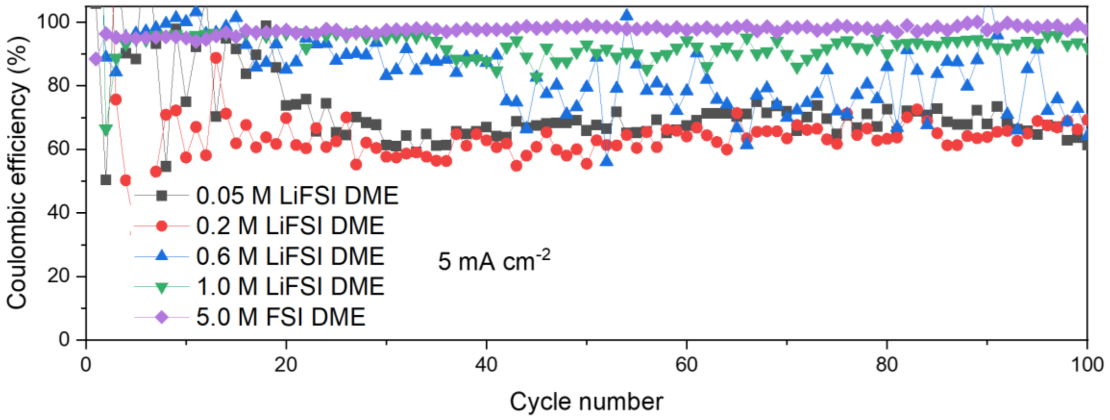

Figure S4. **Cycling performance of Li||Cu cells with different electrolytes at a current density of 5 mA cm^{-2} to an areal capacity of 1 mAh cm^{-2}.**

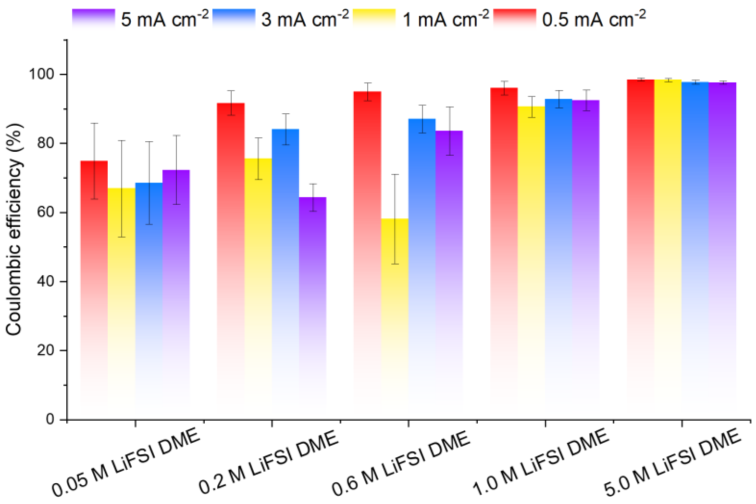

Figure S5. **Average CE of Li||Cu cells during 100 cycles under various current densities.** Li||Cu cells using five different concentration of LiFSI DME electrolytes were tested for 100 cycles at four cycling rates. In all cases, Li was plated on Cu current collector to a capacity of 1mAh cm^{-2}.

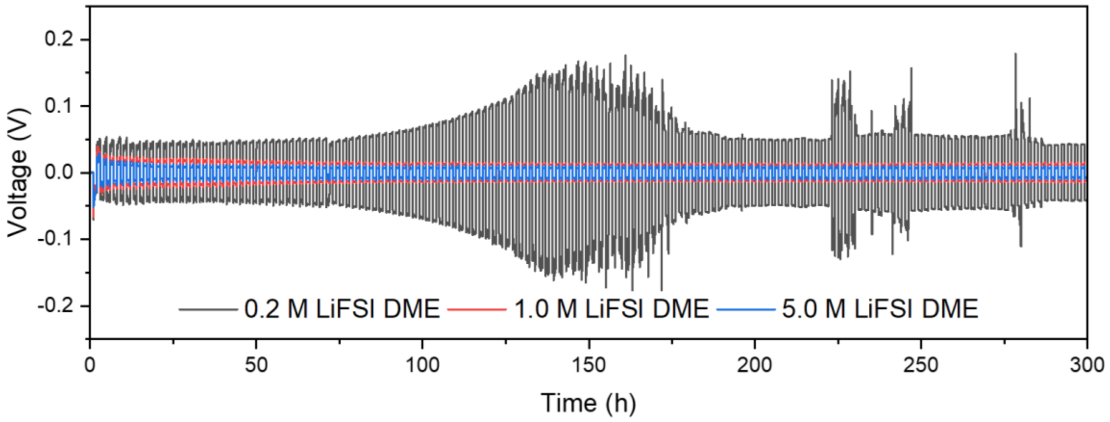

Figure S6. Galvanostatic cycling performance of Li||Li symmetric cells at a current density of 1.0 mA cm^{-2} for 1 h.

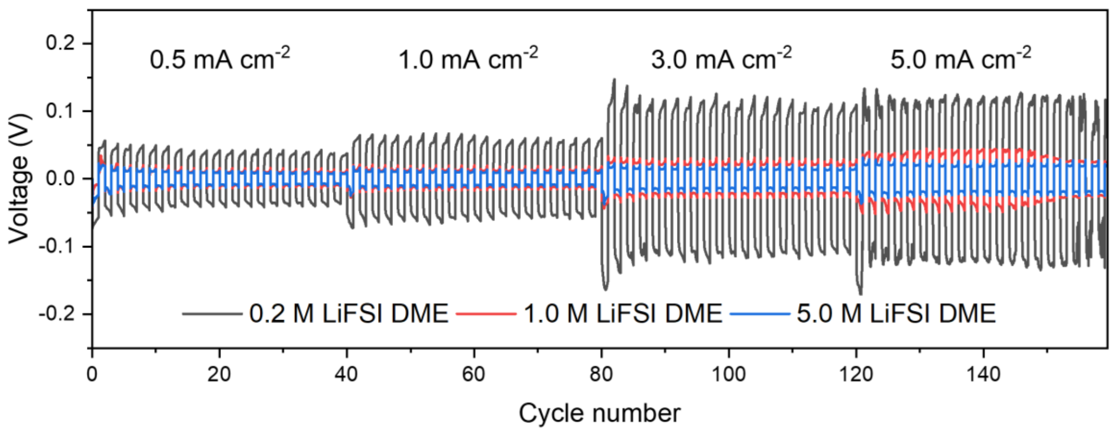

Figure S7. Voltage profile of Li||Li symmetric cells at different current densities from 0.5 mA cm^{-2} to 5 mA cm^{-2}.

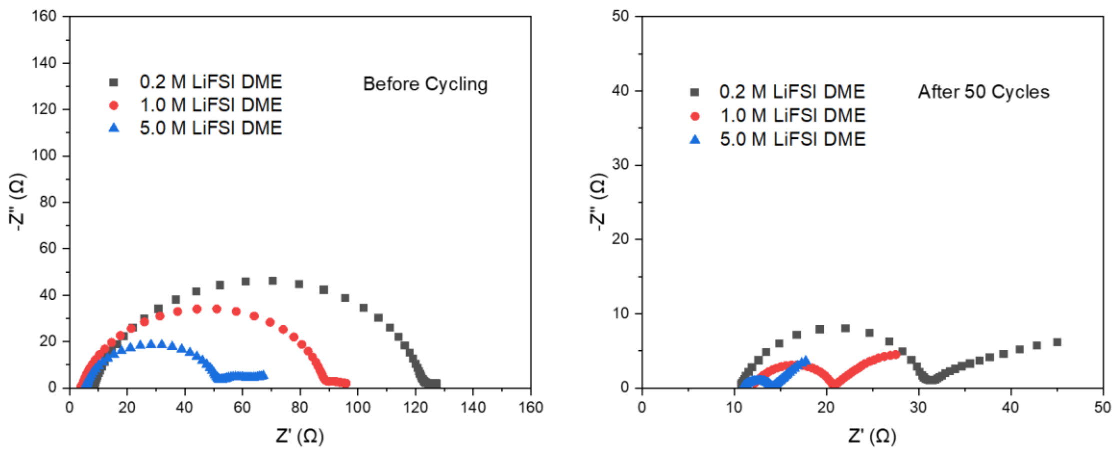

Figure S8. **Electrochemical impedance spectroscopy (EIS) of Li||Li symmetric cells.** Nyquist plots are obtained from the Li||Li symmetric cells using different electrolytes before (left) and after 50 cycles at the current density of 1.0 mA cm^{-2} (right).

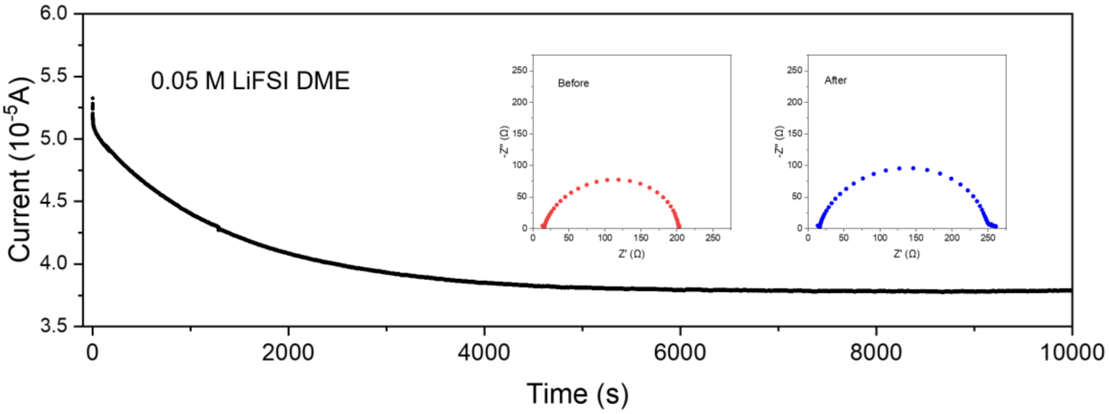

Figure S9. **The chronoamperometry profile of a Li||Li symmetric cell using a 0.05 M LiFSI DME electrolyte under a polarization potential of 10 mV.** Inserts are the corresponding EIS plots before and after polarization, showing the initial and steady-state values of resistance, respectively.

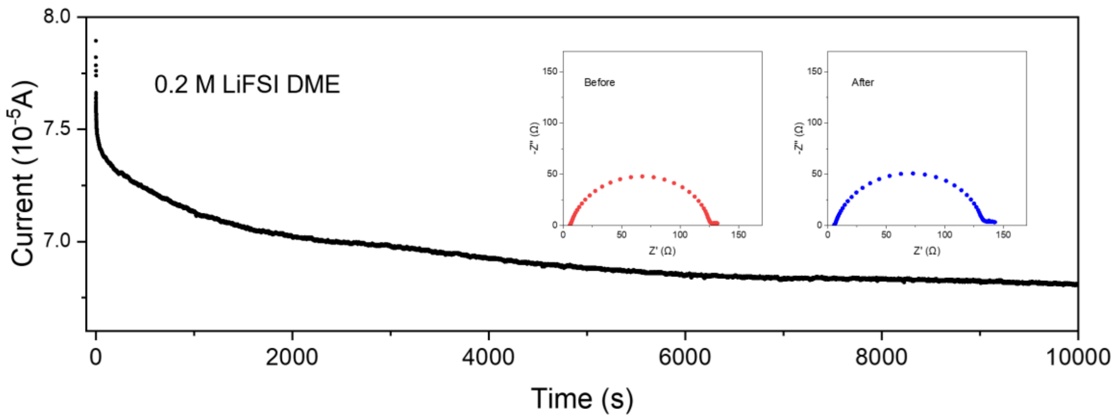

Figure S10. **The chronoamperometry profile of a Li||Li symmetric cell using a 0.2 M LiFSI DME electrolyte under a polarization potential of 10 mV.** Inserts are the corresponding EIS plots before and after polarization, showing the initial and steady-state values of resistance, respectively.

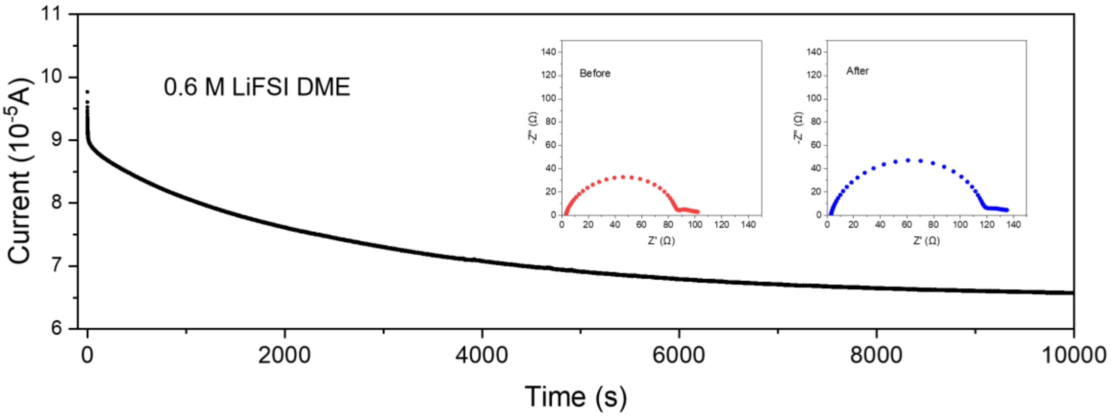

Figure S11. **The chronoamperometry profile of a Li||Li symmetric cell using a 0.6 M LiFSI DME electrolyte under a polarization potential of 10 mV.** Inserts are the corresponding EIS plots before and after polarization, showing the initial and steady-state values of resistance, respectively.

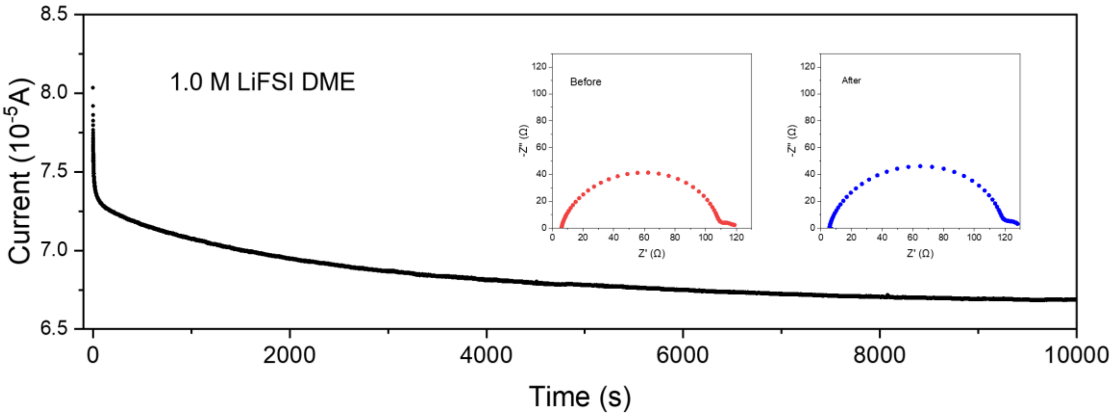

Figure S12. **The chronoamperometry profile of a Li||Li symmetric cell using a 1.0 M LiFSI DME electrolyte under a polarization potential of 10 mV.** Inserts are the corresponding EIS plots before and after polarization, showing the initial and steady-state values of resistance, respectively.

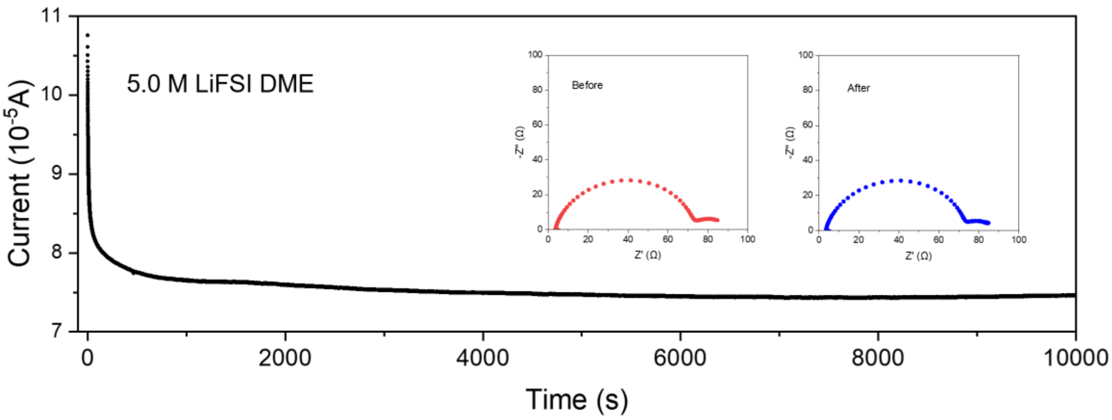

Figure S13. **The chronoamperometry profile of a Li||Li symmetric cell using a 5.0 M LiFSI DME electrolyte under a polarization potential of 10 mV.** Inserts are the corresponding EIS plots before and after polarization, showing the initial and steady-state values of resistance, respectively.

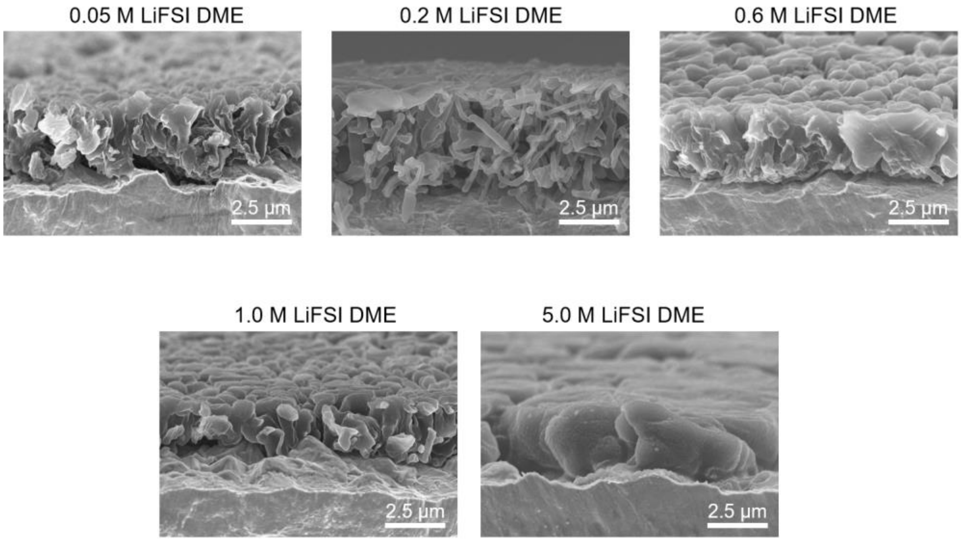

Figure S14. **Cross-sectional SEM images of Li metal microstructures on Cu current collector.**

The current density for Li plating is 0.5 mA cm^{-2} and the deposition capacity is 1 mAh cm^{-2}.

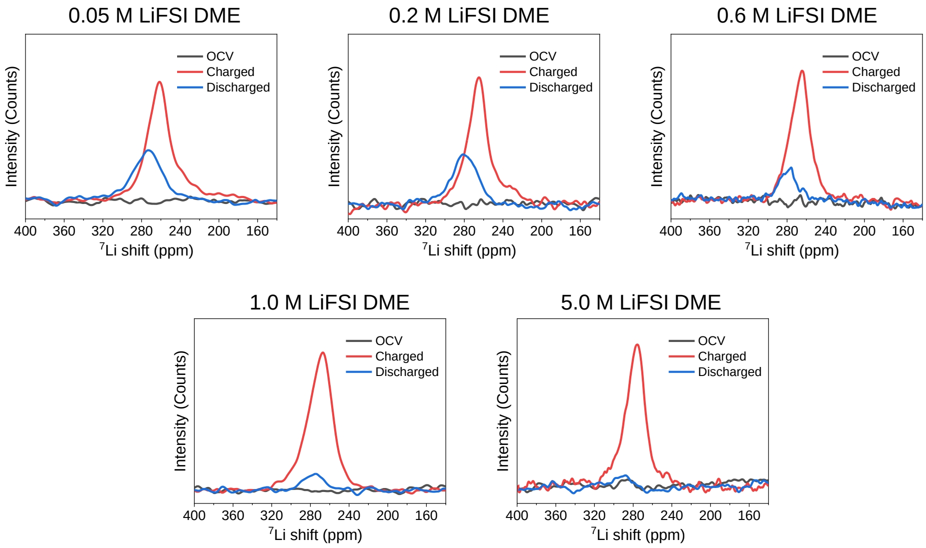

Figure S15. **The spectra extracted from operando ^{7}Li NMR dataset.** Comparison of the Li-metal resonance in the ^{7}Li NMR spectra from the Cu||$LiFePO_4$ cells before (pristine) and after Li plating (charged), and after Li stripping (discharged).

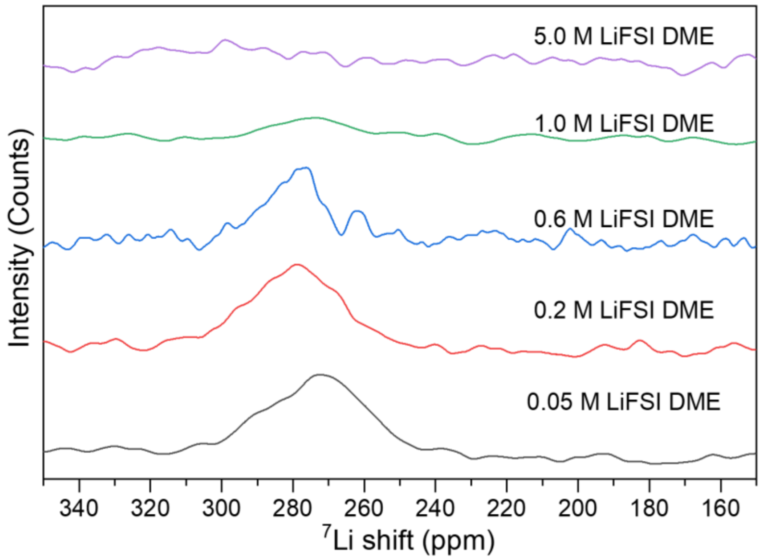

Figure S16. **^{7}Li NMR spectra showing "dead" Li formed in different electrolytes.** The ^{7}Li NMR spectra showing the "dead" Li amount based on the difference between the discharged state and the pristine state in each concentration electrolyte.

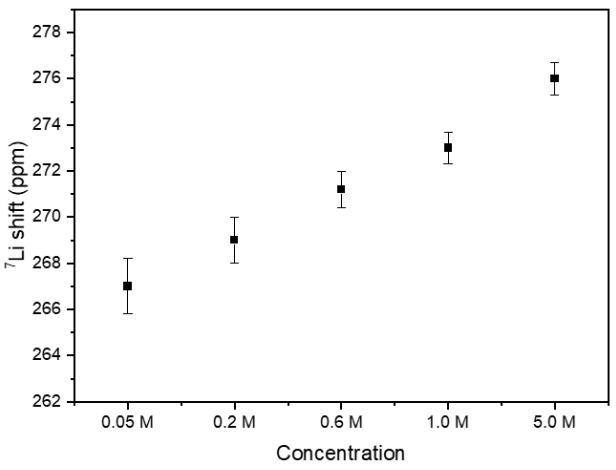

Figure S17. Initial ^{7}Li chemical shift as the function of electrolyte molarity.

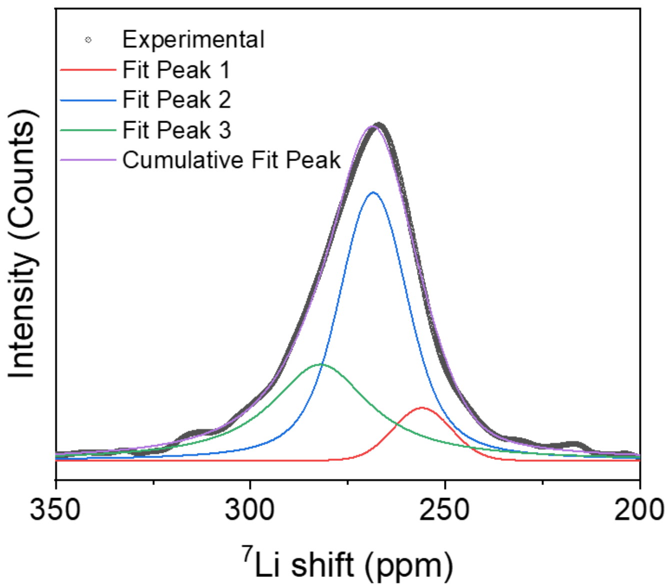

Figure S18. **The example of the fitted spectra of ^{7}Li NMR at the end of Li plating in the first cycle.** The peaks 1, 2 and 3 are indicated with different colors.

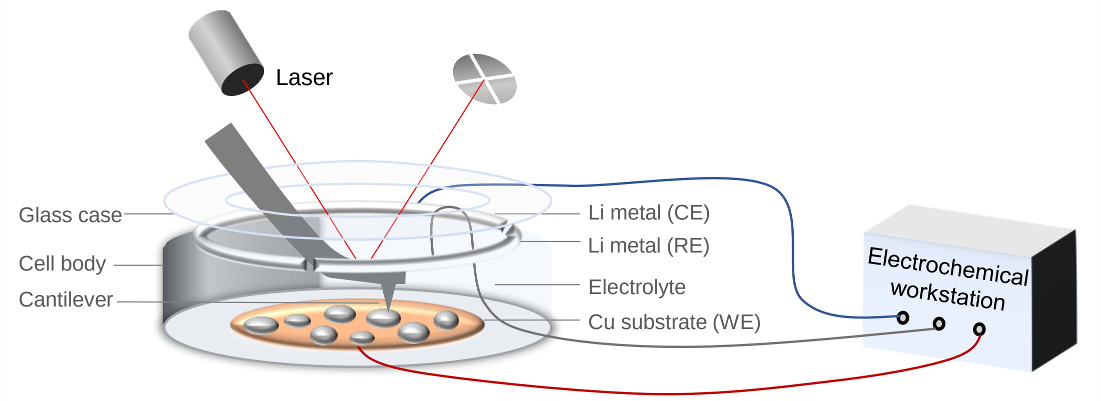

Figure S19. **Schematic illustration of the setup for in-situ electrochemical AFM.** A three-electrode cell is assembled in an argon-filled glove box with a Cu foil as the working electrode (WE) and lithium metal strips as both counter electrodes (CE) and reference electrodes (RE). The discharging (Li plating) process is controlled by an electrochemical workstation (CHI760E) outside of the glovebox.

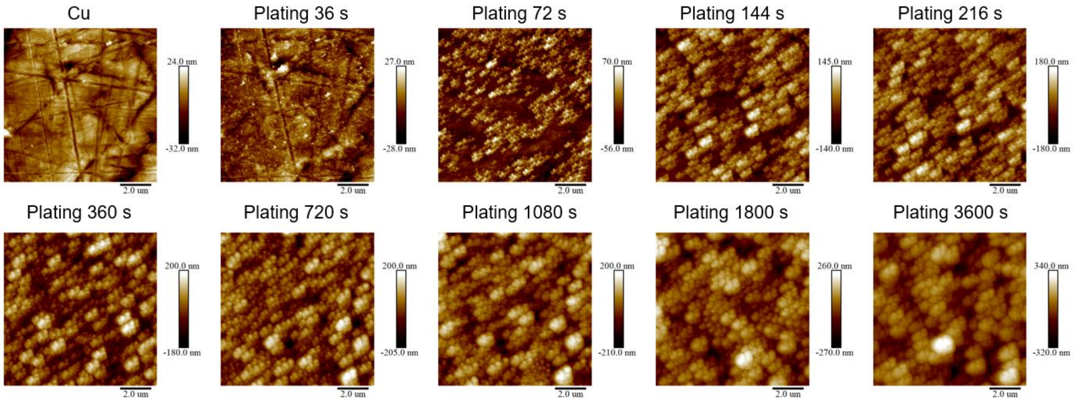

Figure S20. **The in-situ electrochemical AFM images of Li metal deposited on Cu substrate in 0.2 M LiFSI DME electrolyte.**

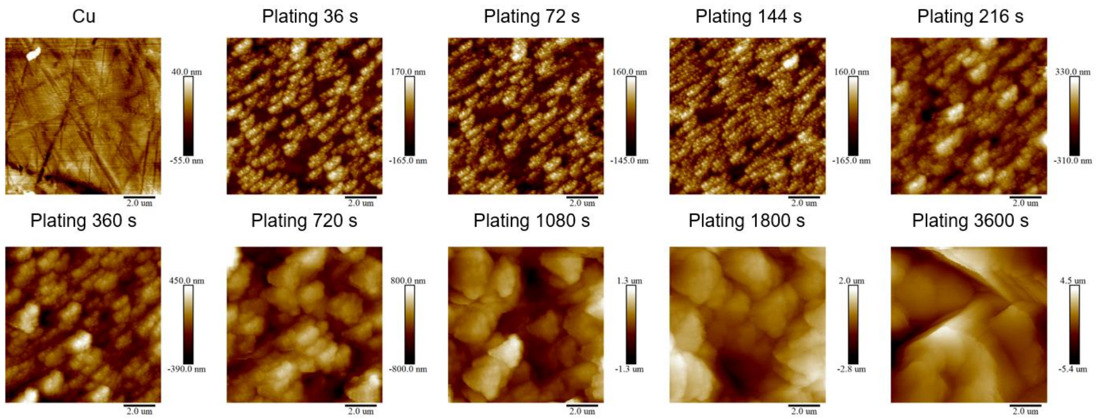

Figure S21. **The in-situ electrochemical AFM images of Li metal deposited on Cu substrate in 1.0 M LiFSI DME electrolyte.**

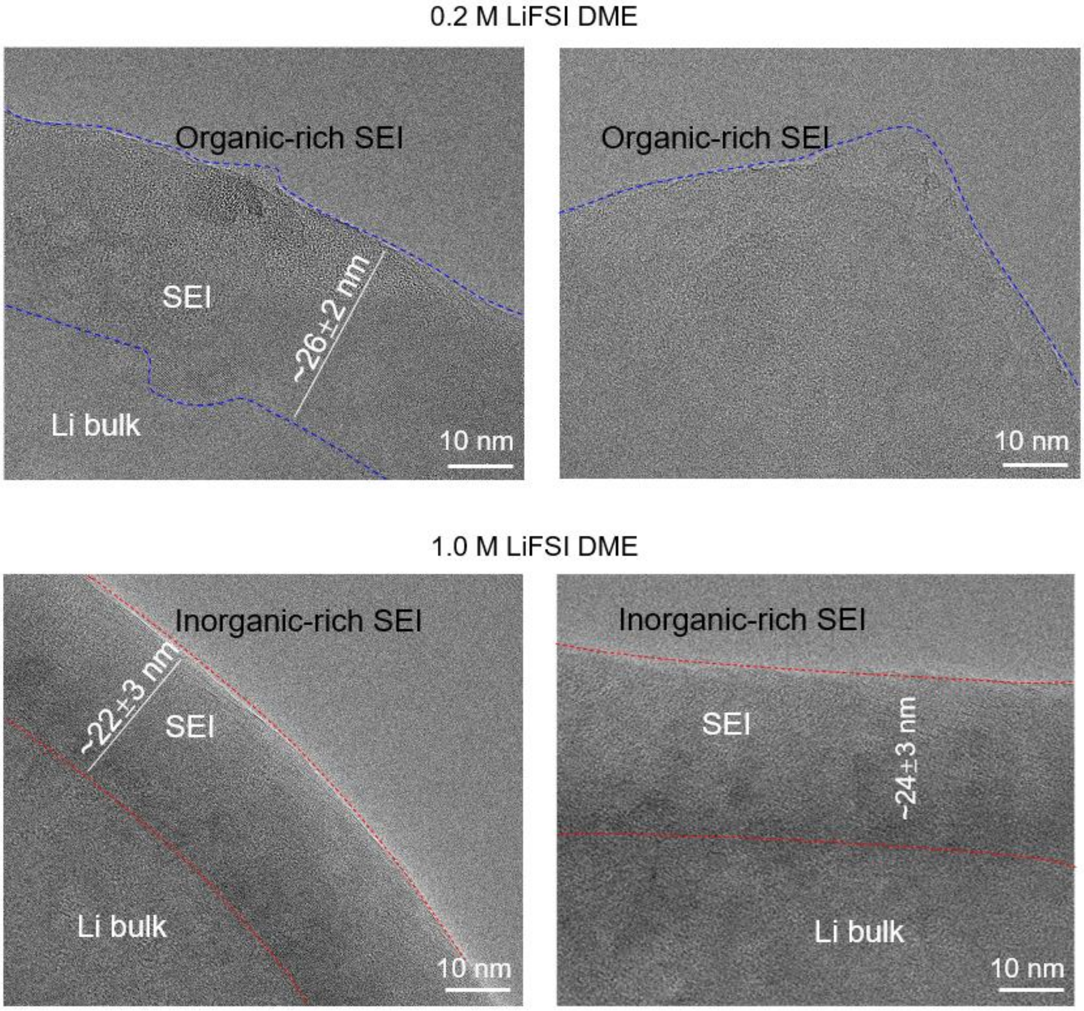

Figure S22. **High-resolution cryo-TEM images of the SEI layer on deposited Li metal in 0.2 M LiFSI DME and 1.0 M LiFSI DME electrolyte.**

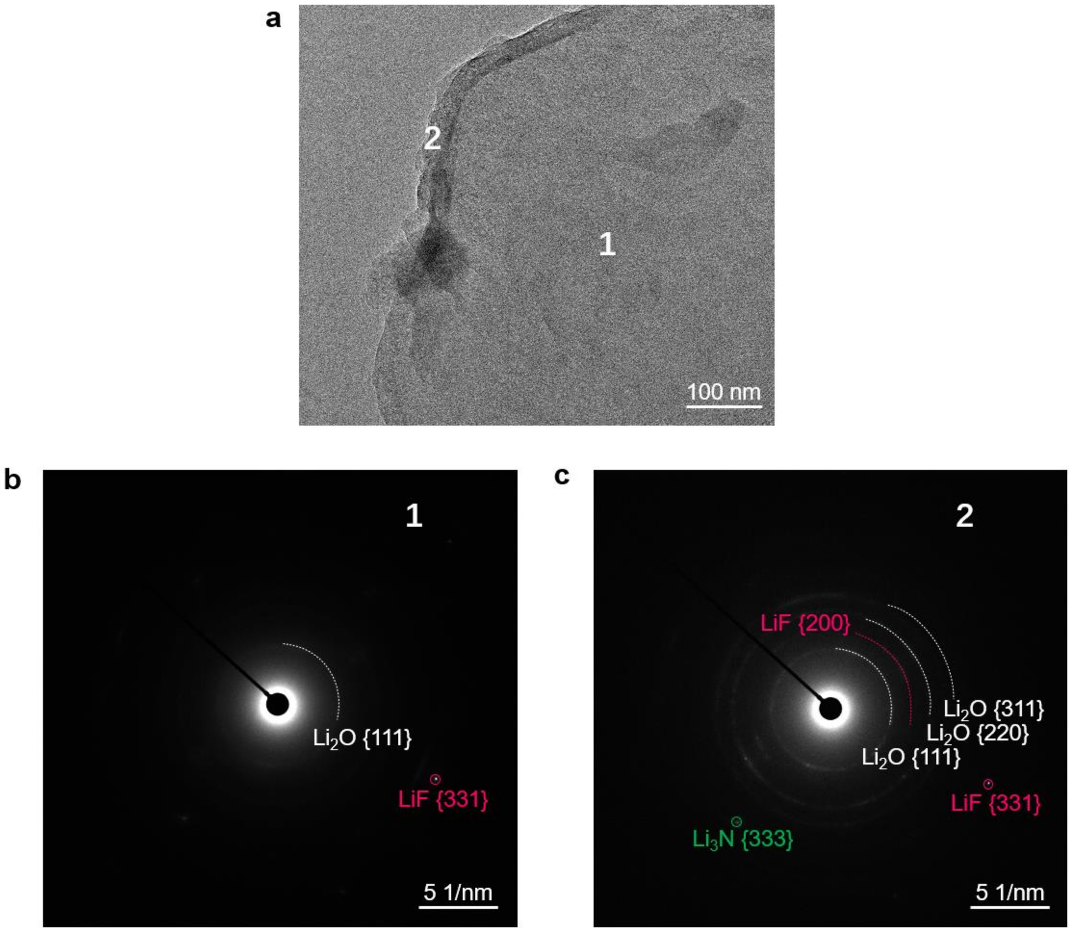

Figure S23. **The selected-area electron diffraction (SEAD) patterns of plated Li metal in 0.2 M LiFSI DME electrolyte. a**, High-resolution cryo-TEM image of the SEI layer on plated Li metal. **b**,**c**, The corresponding SEAD patterns of (**b**) the bulk Li and (**c**) the solid electrolyte interface (SEI) as marked in **a**.

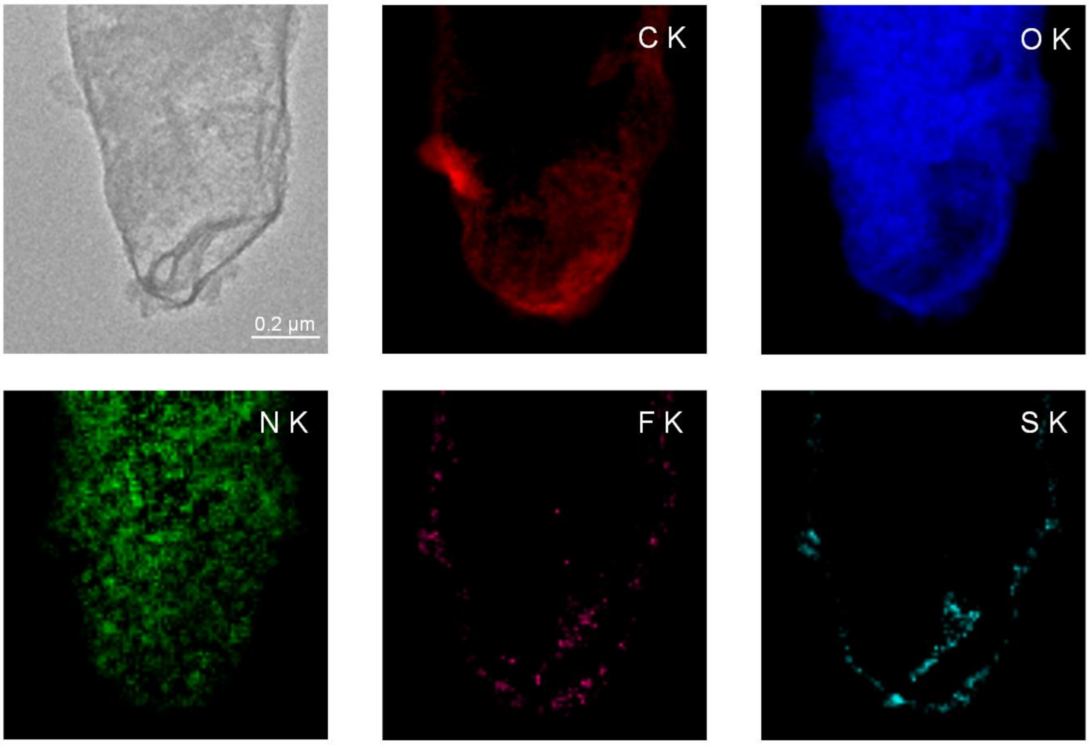

Figure S24. Electron energy loss spectroscopy (EELS) mapping showing the elemental distribution on plated Li metal in a 0.2 M LiFSI DME electrolyte. Representative cryo-STEM ADF image of plated Li metal (top left panel) and corresponding EELS mapping collected on the *K*-edge reveals carbon, oxygen, nitrogen, fluorine and sulfur components.

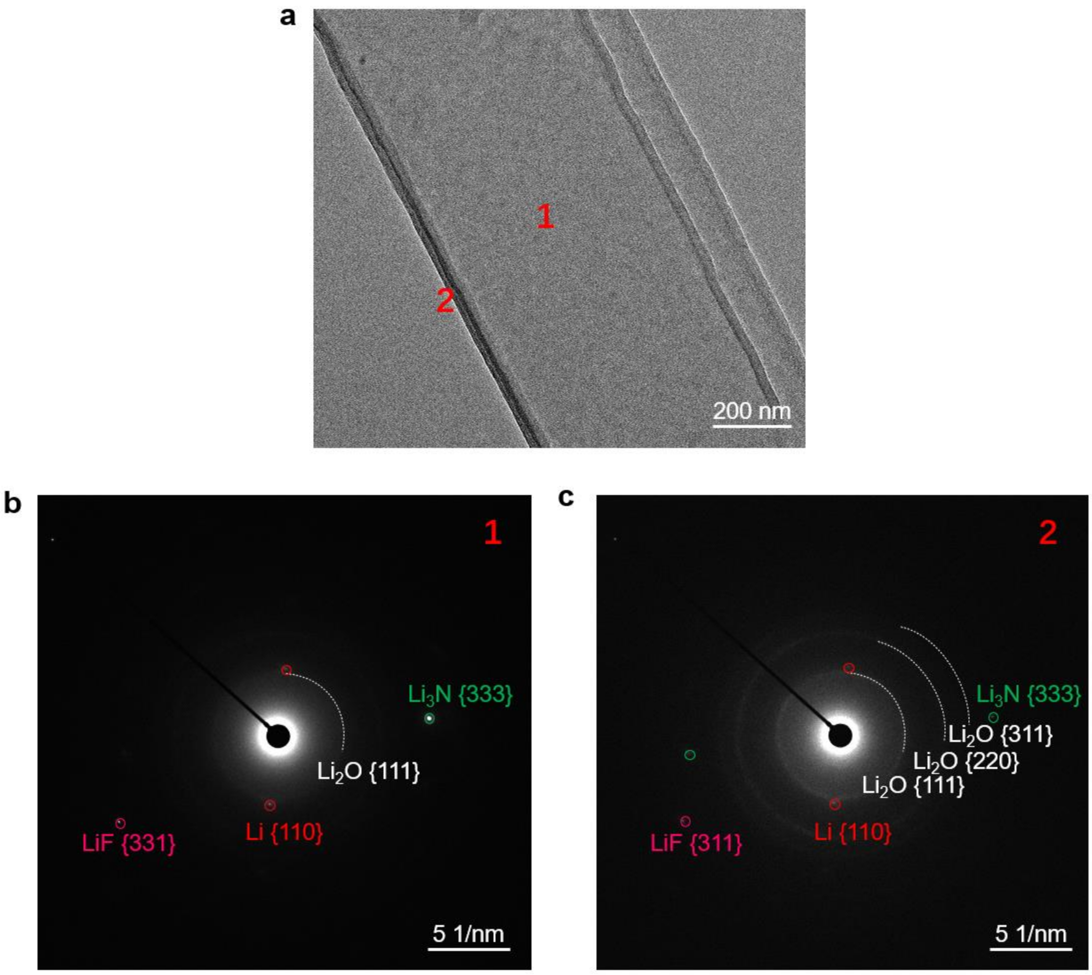

Figure S25. The SEAD pattern of plated Li metal in 1.0 M LiFSI DME electrolyte. a, High-resolution cryo-TEM image of the SEI layer on plated Li metal. **b**,**c**, The corresponding SEAD pattern of (**b**) the bulk Li and (**c**) the SEI as marked in **a**.

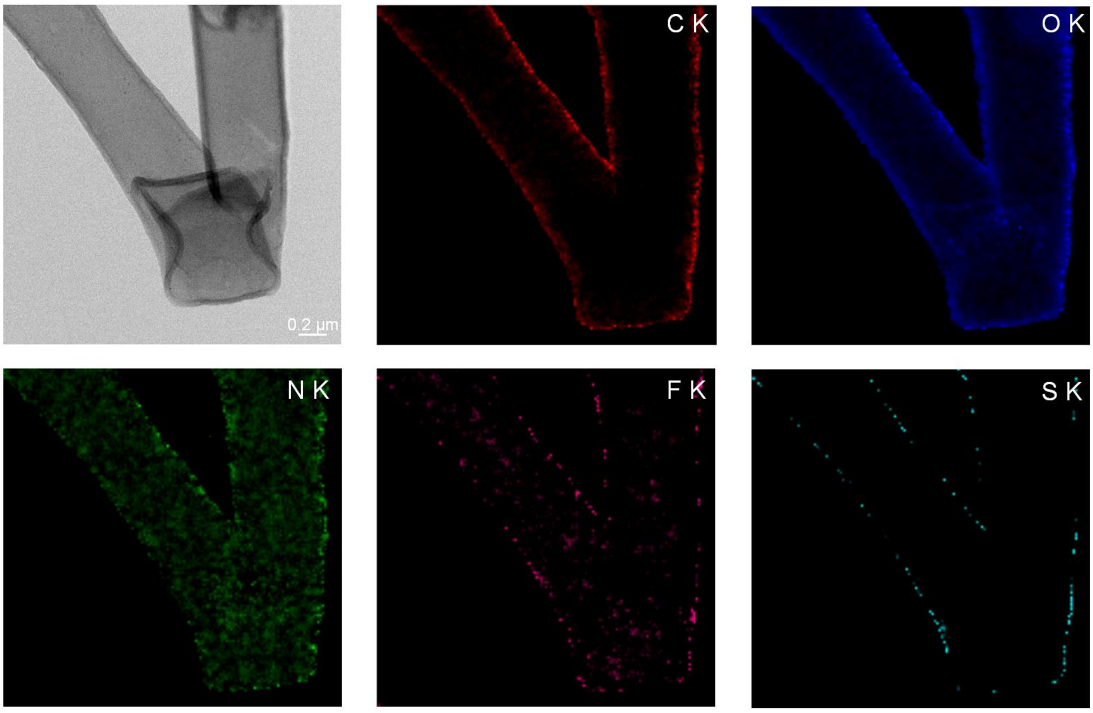

Figure S26. EELS mapping showing the elemental distribution on plated Li metal in a 1.0 M LiFSI DME electrolyte. Representative cryo-STEM ADF image of the deposited Li metal (top left) and corresponding EELS mapping on the *K*-edge reveals carbon, oxygen, nitrogen, fluorine and sulfur components.

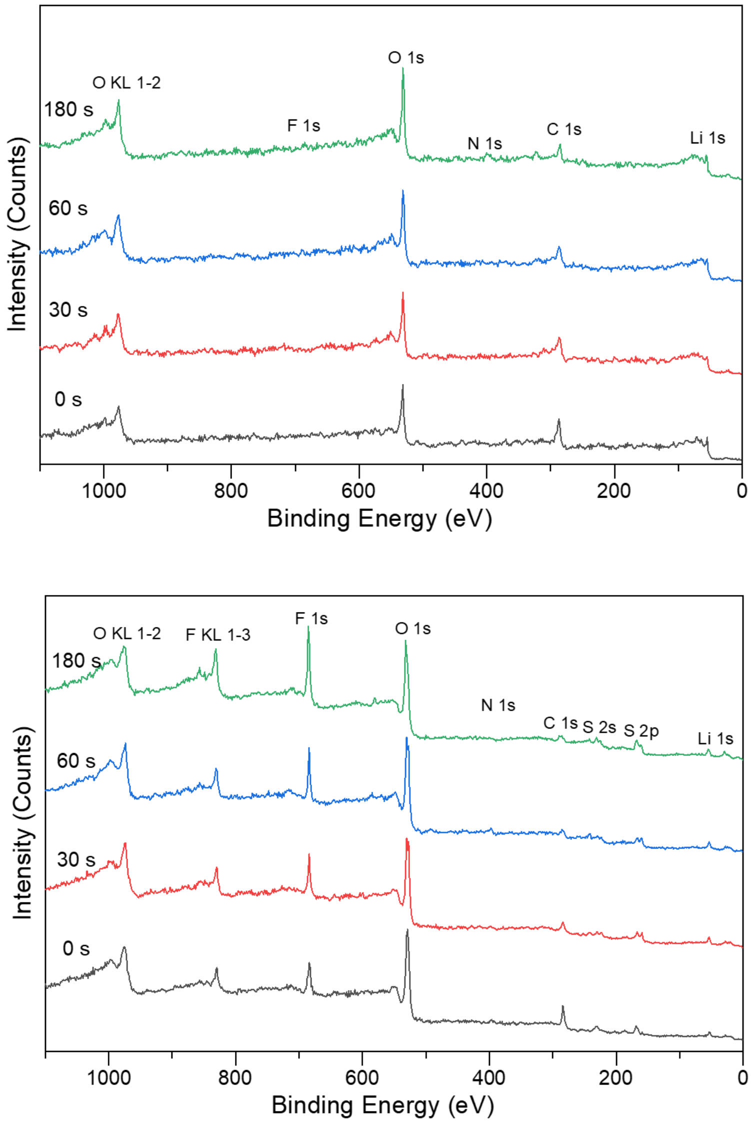

Figure S27. The survey spectra of XPS depth profiles in 0.2 M LiFSI DME (top) and 1.0 M LiFSI DME (bottom) electrolytes.

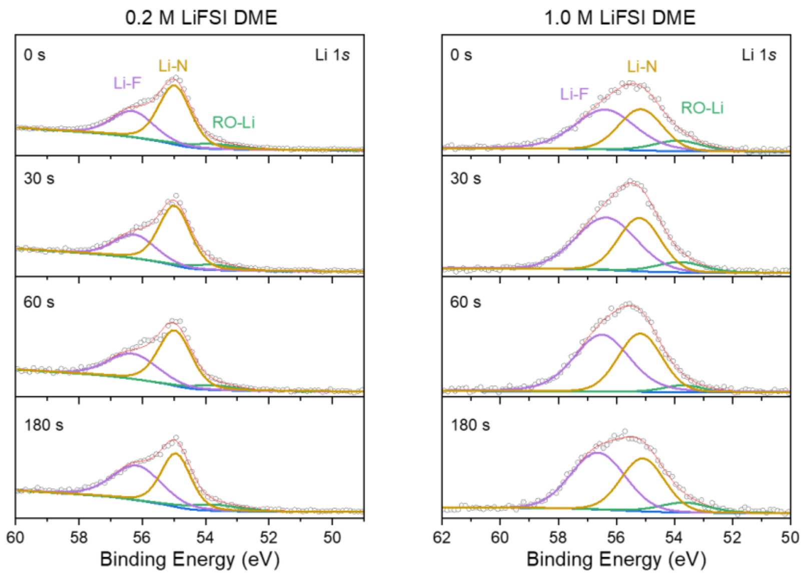

Figure S28. Li 1*s* XPS depth profiles of the SEI formed in a 0.2 M LiFSI DME (left) and a 1.0 M LiFSI DME (right) electrolyte.

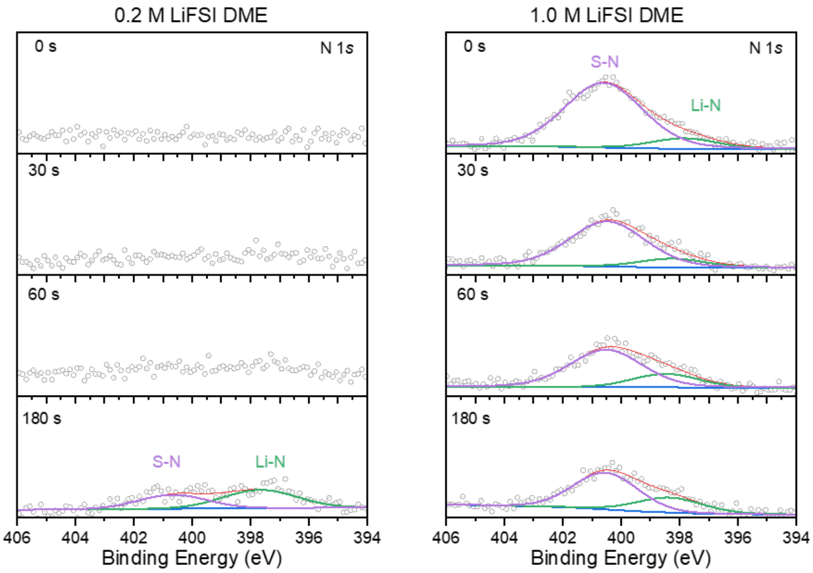

Figure S29. N 1*s* XPS depth profiles of the SEI formed in a 0.2 M LiFSI DME (left) and a 1.0 M LiFSI DME (right) electrolyte.

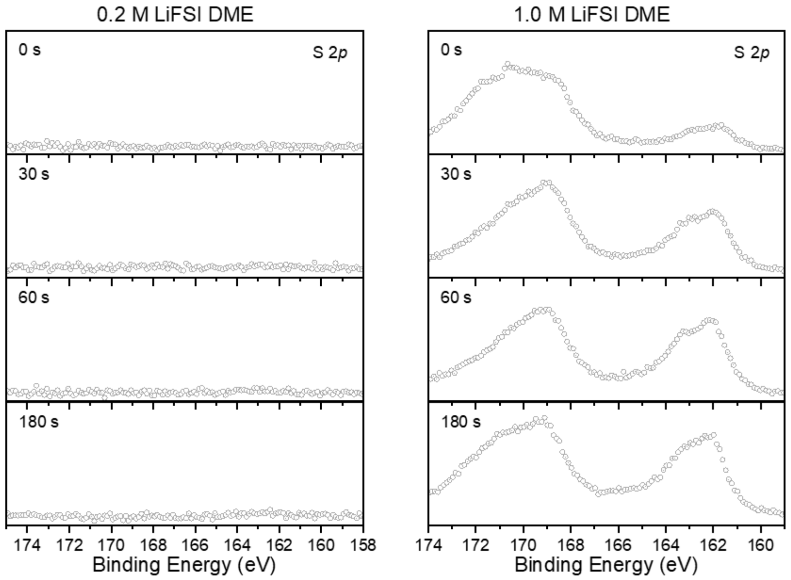

Figure S30. S 2*p* XPS depth profiles of the SEI formed in a 0.2 M LiFSI DME (left) and a 1.0 M LiFSI DME (right) electrolyte.

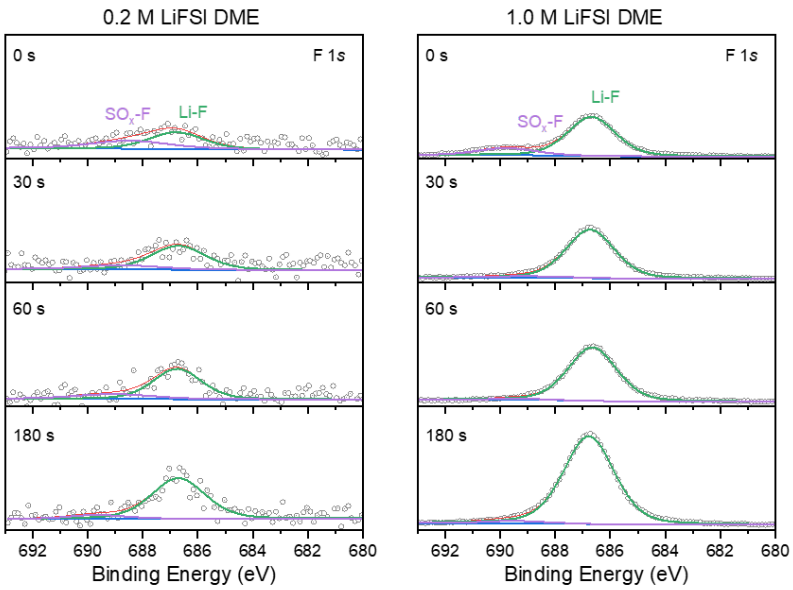

Figure S31. F 1*s* XPS depth profiles of the SEI formed in a 0.2 M LiFSI DME (left) and a 1.0 M LiFSI DME (right) electrolyte.

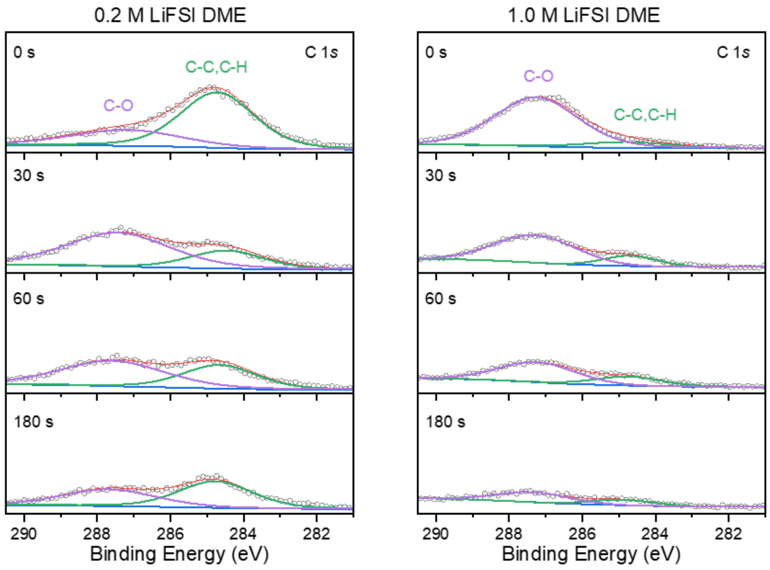

Figure S32. C 1*s* XPS depth profiles of the SEI formed in a 0.2 M LiFSI DME (left) and a 1.0 M LiFSI DME (right) electrolyte.

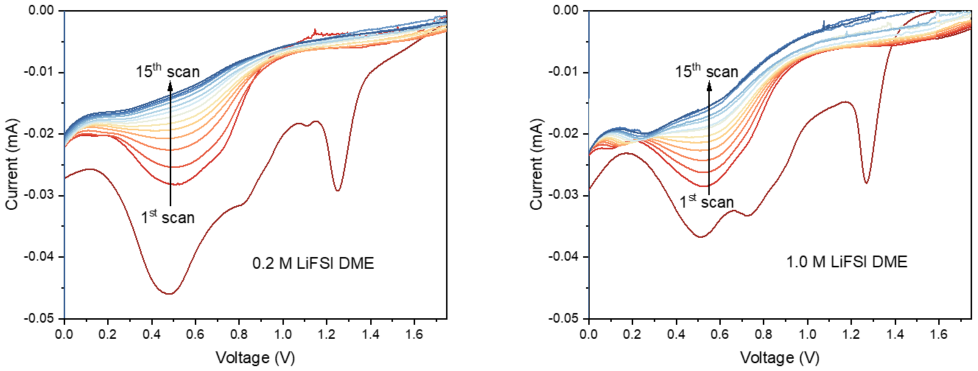

Figure S33. Zoomed-in CV plot of Li plating/stripping in different electrolytes for fifteen cycles with a scan rate of 0.8 mV s^{-1}.

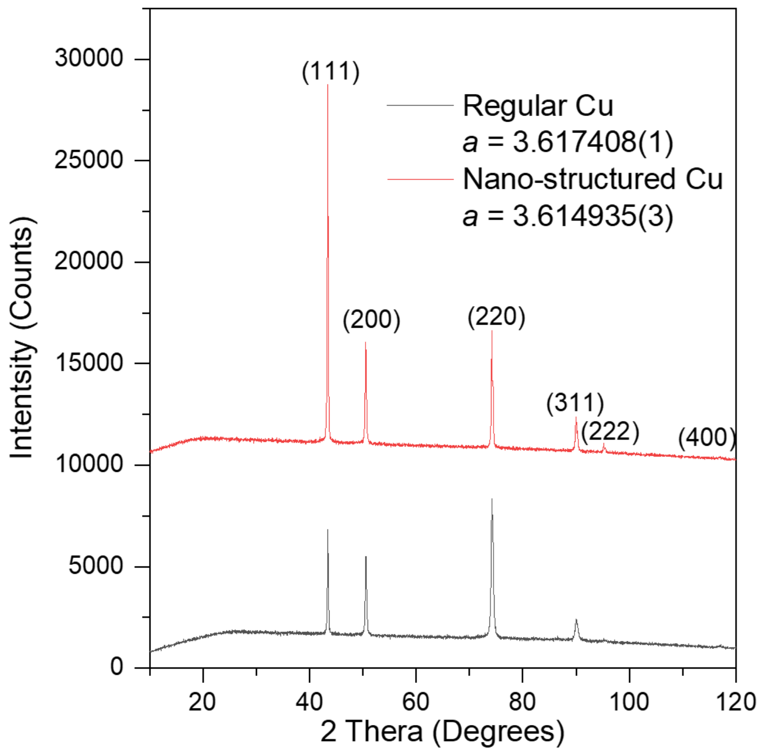

Figure S34. X-ray diffraction patterns of two kinds of Cu foil used in this work. The peaks observed were compared with the standard powder diffraction file of No. 04-0838. Both patterns are indexed in the cubic structure, where the lattice parameters of the Cu foils are almost consistent, but the (111) peak of the nano-structured Cu shows increased intensity.

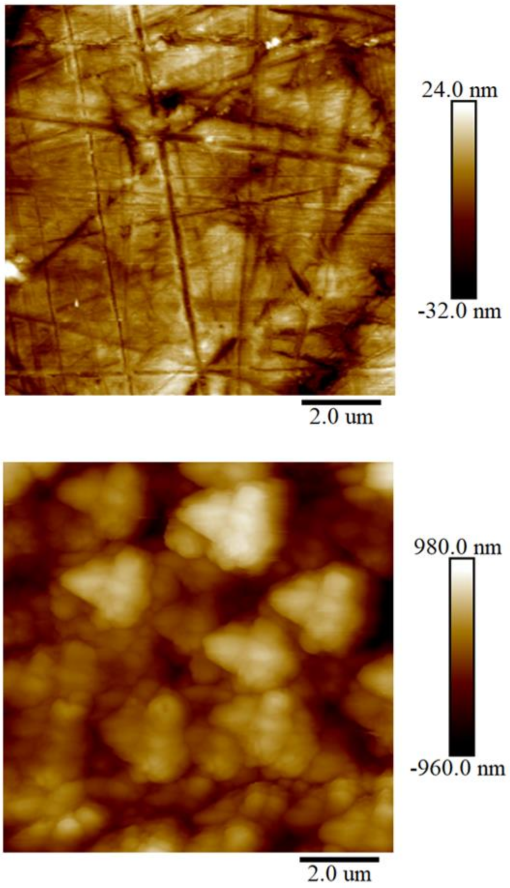

Figure S35. AFM images of the topography of the regular Cu (top) and nano-structured Cu (bottom) in an area of 10 × 10 µm.

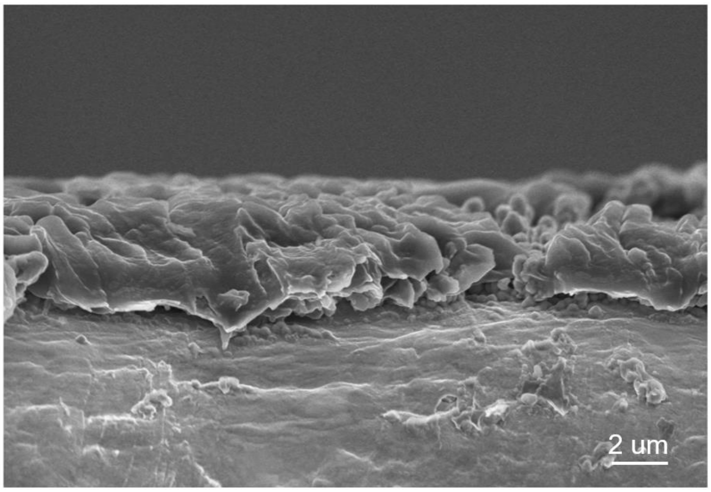

Figure S36. Cross-sectional SEM image of Li deposition on nano-structured Cu foil in a 1.0 M LiFSI DME electrolyte at the current density of 0.5 mA cm^{-2} to an areal capacity of 1 mAh cm^{-2}.

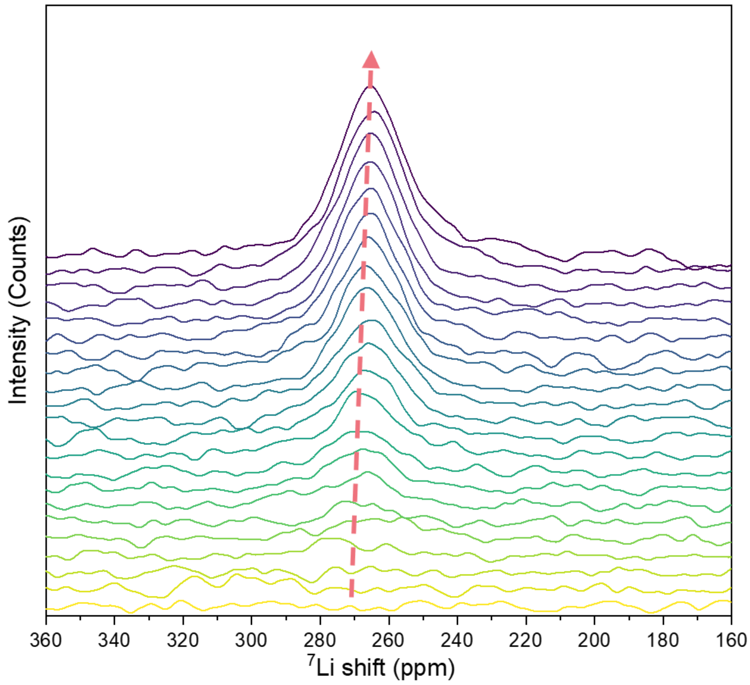

Figure S37. Stack plot of operando ^{7}Li NMR spectra during charging of a Cu|| $LiFePO_4$ cell with nano-structured Cu foil in a 1.0 M LiFSI DME electrolyte.

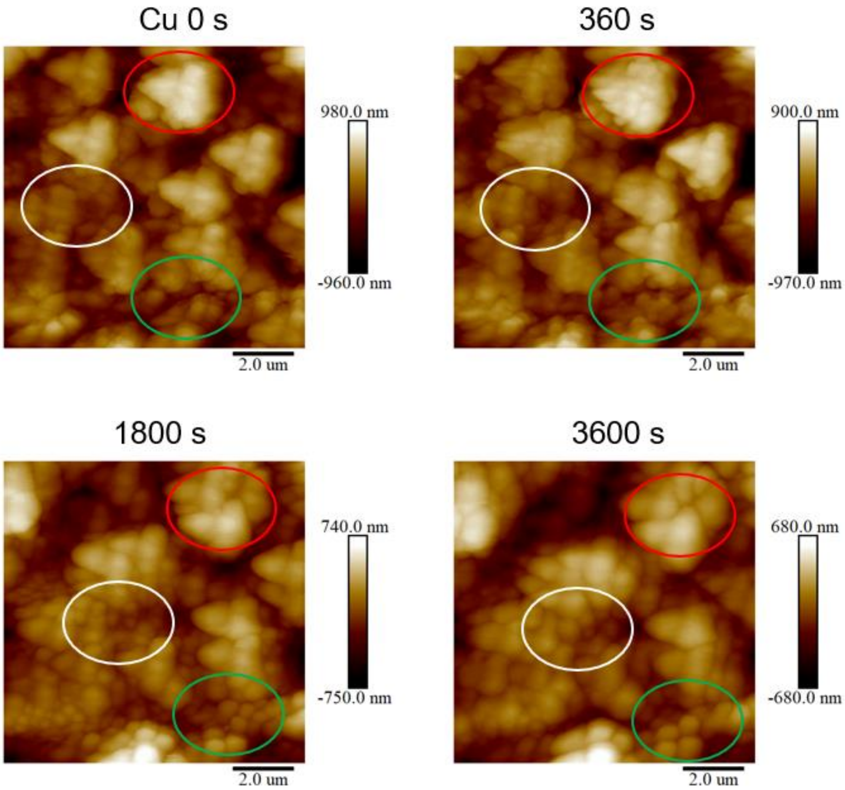

Figure S38. The in-situ electrochemical AFM images of the Cu substrate topography before and after Li plating at 0.5 mA cm^{-2} for 360 s (0.05 mAh cm^{-2}), 1800 s (0.25 mAh cm^{-2}) and 1800 s (0.5 mAh cm^{-2}) on nano-structured Cu foil in 1.0 M LiFSI DME electrolyte. The colored circles with the same size indicate the tomography evolution with different amount of Li metal plating compared to the bare Cu. After plating 360s, new particles can be observed compared with the bare Cu, which is ascribed to the Li deposits. With the capacity of deposited Li increases, some particle coalesced and grow larger.

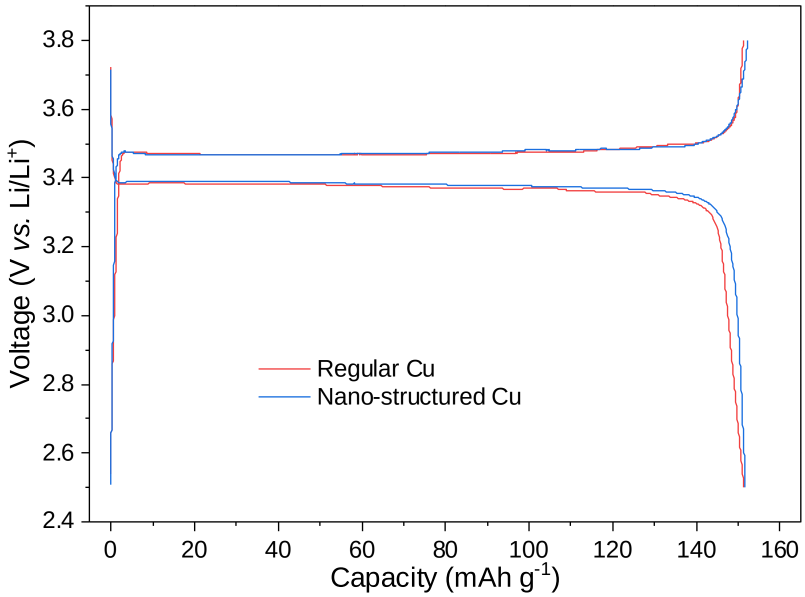

Figure S39. Charge and discharge curves of Cu||$LiFePO_4$ batteries cycled at 1/3C in a 1.0 M LiFSI DME electrolyte with different Cu foils. The areal capacity of $LiFePO_4$ cathode is 2 mAh cm^{-2}. The regular or nano-structured Cu current collector were used to assemble Cu||$LiFePO_4$ after pre-deposit Li of 0.5 times the capacity of cathode electrodes.

Table S1 | Electrochemical and physical properties for LiFSI-DME electrolytes at room temperature.

Molarity of LiFSI in DME (mol L^{-1})	Conductivity (mS cm^{-1})	t_{Li^+}	Viscosity (mPa·s)
0.05	~1.6	0.08±0.03	0.42
0.2	~3.2	0.27±0.05	0.57
0.6	~9.6	0.39±0.03	0.89
1.0	~16.1	0.57±0.04	1.36
5.0	~1.9	0.31±0.05	20.24

Table S2 | Coulombic efficiency, SEI and "dead" Li portion in the first cycle determined by the combination of electrochemical test and operando NMR.

Molarity of LiFSI in DME (mol L^{-1})	Coulombic efficiency (%)	SEI formation capacity (%)	"Dead" Li capacity (%)
0.05	40.3±2.2	16.9±1.3	42.8±2.3
0.2	45.3±1.8	13.7±1.1	41.0±1.9
0.6	66.6±1.6	10.8±1.0	22.5±1.2
1.0	77.7±1.3	9.0±0.9	13.1±0.9
5.0	88.5±1	8.2±0.5	3.3±0.3

Supplementary Note 1

The quantification of the capacity loss is based on the method of Gunnarsdóttir et al.[2]. The SEI formation capacity in the first cycle can be estimated from the dead Li by NMR and the Coulombic efficiency (CE) from the electrochemistry. The capacity loss (CL) in the first cycle is defined as:

$$CL = C_{plating} - C_{stripping} \qquad \text{Equation S3}$$

The coulombic efficiency is calculated as follows:

$$CE = \frac{C_{stripping}}{C_{plating}} \qquad \text{Equation S4}$$

Where the $C_{plating}$ is the full plating capacity (1mAh cm^{-2} in this work), assuming no side reactions. Then the capacity loss (CL) in the first cycle can be rewritten as:

$$CL = C_{plating} \times (1 - CE) \qquad \text{Equation S5}$$

CL include the capacity loss from dead Li formation ($C_{Dead\ Li}$) and capacity loss from SEI formation (C_{SEI}) in electrochemical measurements as follows:

$$CL = C_{Dead\ Li} + C_{SEI} \qquad \text{Equation S6}$$

$C_{Dead\ Li}$ is estimated by the following equation:

$$C_{Dead\ Li} = (C_{Plating} - C_{SEI}) \times \frac{I_{stripping}}{I_{plating}} \qquad \text{Equation S7}$$

Where $\frac{I_{stripping}}{I_{plating}}$ the ratio of the integrated intensity of the Li metal at the end of the 1st discharge to that measured at the end of the 1st charge. Therefore, CL can be calculated Equation S8, and the C_{SEI} and $C_{Dead\ Li}$ can be obtained.

$$CL = \left(C_{Plating} - C_{SEI}\right) \times \frac{I_{stripping}}{I_{plating}} + C_{SEI} \qquad \text{Equation S8}$$

References

1. Abraham, K. M.; Jiang, Z.; Carroll, B., Highly Conductive PEO-like Polymer Electrolytes. *Chemistry of Materials* **1997,** *9* (9), 1978-1988.

2. Gunnarsdóttir, A. B.; Amanchukwu, C. V.; Menkin, S.; Grey, C. P., Noninvasive In Situ NMR Study of "Dead Lithium" Formation and Lithium Corrosion in Full-Cell Lithium Metal Batteries. *Journal of the American Chemical Society* **2020,** *142* (49), 20814-20827.
